# Optical pulling force in carbon nanotubes: Manifestation of non-local conductivity

**DOI:** 10.1016/j.isci.2026.117817

**Published:** 2026-10-13

**Authors:** Tomer Berghaus, Touvia Miloh, Gregory Ya. Slepyan

**Affiliations:** 1 School of Mechanical Engineering, Tel Aviv University, Tel Aviv 69978, Israel; 2 School of Electrical Engineering, Tel Aviv University, Tel Aviv 69978, Israel

**Keywords:** optical pulling force, spatial dispersion, edge effects, carbon nanotubes

## Abstract

We develop a new theory of an optical force exerted on a carbon nanotube (CNT) with a non-local conductivity. To determine the optical force, we employ an integral-equation-based approach in terms of the current density. The analysis is constructed for a finite-length cylindrical CNT by rigorously accounting for edge effects. In addition to numerical solutions of the integral equation, we obtain an approximate analytical expression for the optical force acting on the CNT, which shows good agreement with numerical simulations. We also demonstrate the existence of some frequency ranges in which the optical force becomes negative, corresponding to the optical pulling effect. Such a pulling behavior is shown to originate from the non-locality of the conductivity. This work advances theoretical understanding of optomechanical interactions in low-dimensional conductors and clarifies the role of spatial dispersion in the emergence of optical pulling forces.

## Highlights

• Finite CNTs exhibit negative axial optical forces under oblique plane-wave incidence
• Spatial dispersion (non-local conductivity) is essential for optical pulling in CNTs
• Forward-scattered momentum exceeds combined incident and backward-scattered momenta

## Graphical abstract

**Figure:**
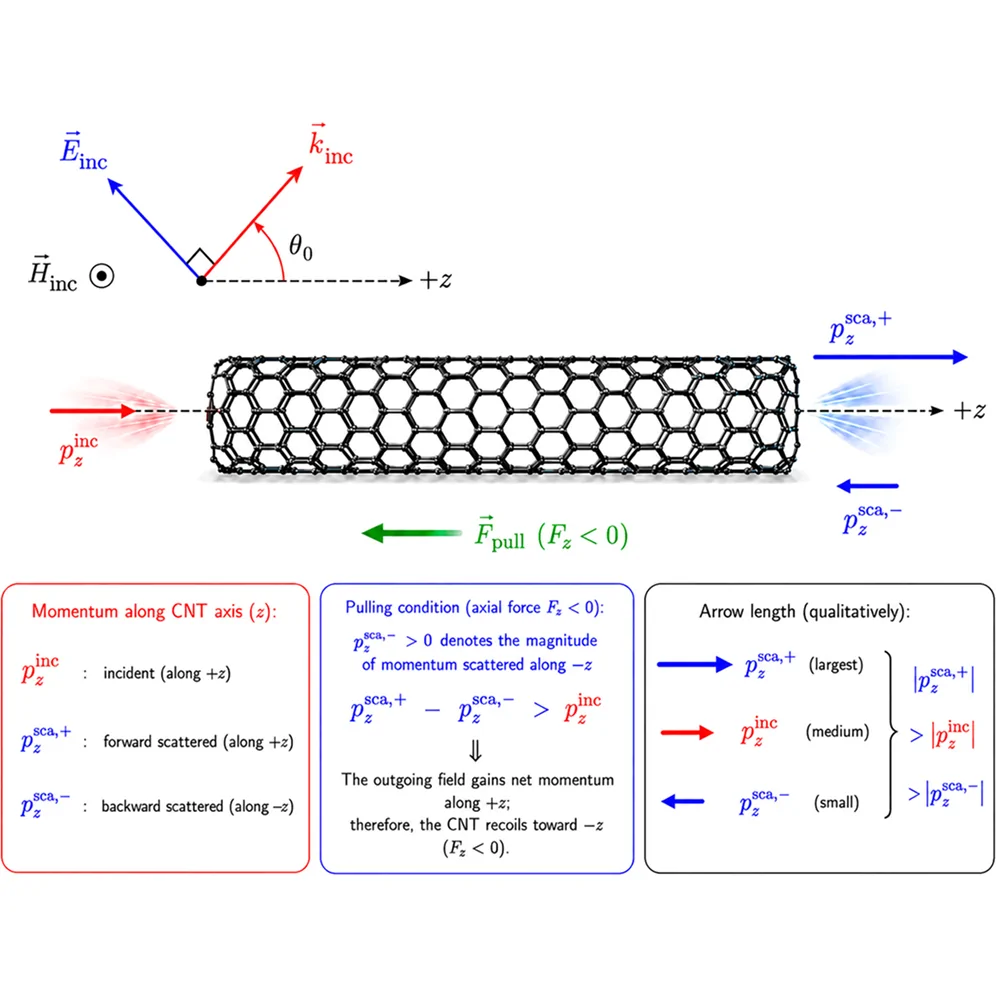

## Teaser

Optics; Condensed matter physics; Applied sciences; Photonics

## Introduction

Studying forces and torques arising from the interaction of light with matter is essential for understanding fundamental aspects of electrodynamics and has opened the way to a broad range of non-destructive and non-contact manipulation techniques for micro- and nano-scale particles in physics,^1^^,^^2^^,^^3^^,^^4^^,^^5^^,^^6^^,^^7^^,^^8^^,^^9^^,^^10^^,^^11^^,^^12^ biology,^6^ chemistry,^2^^,^^13^ and medicine.^14^ One of the earliest milestones in this field were the pioneering observations of electromagnetic radiation pressure by Lebedev^15^ and Nichols with Hull^16^ (both in 1901). The modern development of this research area can naturally be divided into two principal stages. The first stage began with Ashkin’s pioneering works,^17^^,^^18^ which focused on micrometer-sized dielectric or metallic particles manipulated by strongly focused laser beams in the visible frequency range. During this period, theoretical models of optical forces were developed within the realm of classical electrodynamics. Material response was described phenomenologically by constitutive parameters such as permittivity and conductivity. Typically, the particle size was much smaller than the wavelength, and scattering was treated in the Rayleigh regime by employing the dipole approximation.

Within this framework, two fundamental mechanisms of optical forces were identified. The first is the gradient force, governed by the spatial gradient of the field intensity, which is present even in lossless particles.^17^ The second is the scattering force, associated with momentum transfer from the electromagnetic field, and exists in absorbing media.^17^ In most conventional configurations, optical forces act in the direction of wave propagation (optical pushing). One of the practical outcomes of this stage was the establishment of optical tweezers^13^^,^^19^^,^^20^^,^^21^^,^^22^^,^^23^ and modern force microscopy techniques.^24^

Recent advances in nanotechnology have initiated a second stage in the physics of optical forces. First, the accessible frequency range was expanded toward the infrared and terahertz domains. Second, the range of physical mechanisms governing optical forces has been significantly broadened. At the nanoscale, the origins of optical forces are no longer reducible solely to Lorentz polarization of atomic ensembles or Ohmic conduction of free electrons in a crystal lattice. In many cases, it is often insufficient to employ a purely local classical description, expressed in terms of effective permittivity and permeability.

The conventional picture has therefore been extended to include effects of chirality,^25^^,^^26^ plasmonic resonances in noble metals,^27^^,^^28^ optical gain and gain-loss balance within a single particle,^29^^,^^30^ spatial dispersion^31^ (non-locality of current or dipole response at a given point), composite configurations such as Janus particles,^32^^,^^33^ excitonic effects in semiconductor nanostructures,^34^^,^^35^ optical nonlinearities,^36^ metamaterials,^37^^,^^38^ and spinning particles.^39^ A major conceptual advance of this stage was the prediction and realization of counterintuitive negative optical forces acting in the opposite direction of wave propagation^40^^,^^41^^,^^42^^,^^43^^,^^44^^,^^45^^,^^46^^,^^47^^,^^48^^,^^49^^,^^50^^,^^51^ (optical pulling forces). A negative optical torque was also predicted.^52^ From a practical perspective, these new developments have extended the use of optical manipulation techniques into lower-frequency regimes as well as to a wide range of novel nanoscale systems.

Despite the substantial progress, several fundamental issues remain unresolved. Optical forces are commonly calculated by integrating the electromagnetic Maxwell stress tensor (MST) over a closed surface enclosing the object.^53^ There exist several formulations for the macroscopic electromagnetic stress tensor.^54^^,^^55^^,^^56^^,^^57^^,^^58^ While these formulations yield identical results when the integration surface lies entirely in a homogeneous, unbounded medium, they can lead to different predictions when the surface intersects material boundaries, such as those of condensed matter.^59^ The selection of the appropriate tensor formulation in such cases remains an open question.

Another common assumption is the use of a point-like scatterer model, in which the incident field is considered practically unperturbed by the presence of the particle (Rayleigh’s approximation). However, the validity of this approximation is not always guaranteed, even when the particle size is small compared to the wavelength.^51^ Attempts to refine the theory within the Rayleigh regime, by accounting, for example, for the longitudinal field component in tightly focused beams, have been reported.^60^

In recent years, growing attention has been devoted to optical forces in carbon-based nanostructures, such as graphene and carbon nanotubes (CNTs), owing to their exceptional electronic and optical properties.^61^^,^^62^^,^^63^^,^^64^^,^^65^^,^^66^^,^^67^^,^^68^ In this work, we present an original study of optical forces acting on a CNT beyond the conventional dipole approximation, by taking into account the effect of the non-local surface conductivity. Thus, the physical nature of the pulling mechanism under consideration is fundamentally different from that known in several other nanostructures (Janus particles,^32^^,^^33^ metamaterials,^37^^,^^38^ and plasmonic nanorods^19^). We demonstrate that a realistic description of the problem requires considering a finite-length CNT model, which introduces the fundamental challenge of accurately capturing edge (end) effects. Within this framework, we also demonstrate the emergence of an optical pulling nature in CNTs, which originates from the non-local nature of the conductivity and which has no analog in the traditional treatment of locally responding structures.

## Results

### Non-local CNT conductivity

The model of non-local conductivity of a CNT was developed previously,^69^ covering the wide frequency range (from THz to the visible range). We consider a finite-length conductive zigzag CNT (*m*,0) with *m* = 3*s*, where *s* is an integer value (Figure 1) and simply employ the Kubo formalism for low-energy excitations, constrained by the condition *E* < 1 eV. For this purpose, we adapt it for the case of a pseudo-spin electron liquid, in a similar manner to the approach used for a monolayer graphene in previous works.^70^^,^^71^ To this end, we consider the electronic states near the K-points, assuming a linear approximation for *ε*_**p**_ = ±*v*_*F*_|**p** − **p**_*F*_|(*v*_*F*_ is the Fermi velocity). In addition, we consider a collision-free electron motion for low temperatures (*T* ≪ *μ*, *μ* is the electrochemical potential). In contrast to graphene,^70^^,^^71^ for a CNT we also consider the transverse electron confinement (i.e., the azimuthal component of the electron momentum is discrete while the longitudinal component is continuous).

**Figure 1:**
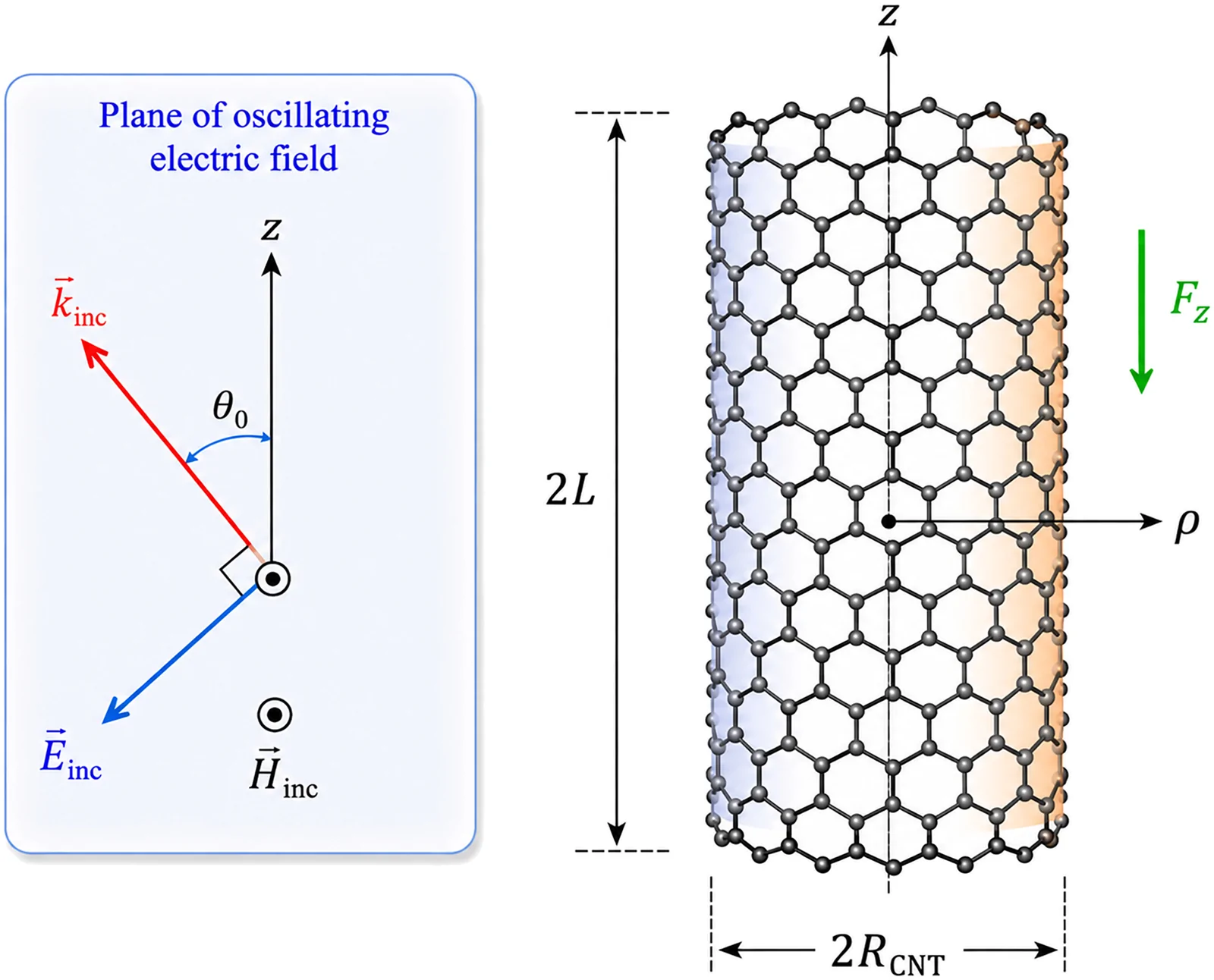
Configuration of a zigzag CNT with unclosed ends CNT is excited by plane wave with angle of incidence *θ*_0_ counted from the longitudinal axis. The origin of cylindrical coordinate system is placed at the central point of CNT axis. The dominative component of the optical force is axial one (it is shown the direction of pulling case).

The electromagnetic fields and the induced currents in the case of spatial dispersion can be expressed as a superposition of traveling waves *exp*[*i*(*qz* − *ωt*)] with wavenumber *q*, which are propagating in opposite directions. Far from the CNT terminations (ends), their interaction can be accurately modeled by using the corresponding conventional infinite-length CNT approximation. Reflections from the extreme ends of the CNT and accounting for non-local effects are incorporated in the present formulation, by applying the so-called “additional” boundary conditions^72^ (for detailed calculations see Supplemental Material^73^).

The conductivity in momentum space can be defined as *σ*_*zz*_(*ω*, *q*) = *i*[Π(*ω*, *q*) − Π(0, *q*)]/*ω*, where

(Equation 1) $$\Pi \left(\omega ,q\right)=\sum_{mn}\sum_{p}\frac{F\left(\varepsilon_{m,p+q}\right)-F\left(\varepsilon_{np}\right)}{\left[\varepsilon_{np}-\varepsilon_{m,p+q}-\hbar \left(\omega +i0\right)\right]}{\left|{\left\langle {\hat{j}}_{z}\right\rangle }_{mn,p,p+q}\right|}^{2}\text{,}$$

denotes the polarizability and ${\left\langle {\hat{j}}_{z}\right\rangle }_{mn,p,p+q}$ represent the matrix elements of the operators of the current density. The symbol *ω* + *i*0 is conventionally associated with collision-less attenuation. The integer summation over *p* is a superposition over the states with different momenta and is a conventional form of indicating an integration over the Brillouin zone. It is also convenient to split the total non-local conductivity into inter and intra components, namely, $\sigma_{zz}\left(\omega ,q\right)=\sigma_{zz}^{\text{inter}}\left(\omega ,q\right)+\sigma_{zz}^{\text{intra}}\left(\omega ,q\right)$. The first term with m ≠ n in 1, corresponds to interband transitions while the second to intraband transitions (also known as the Drude component in conductivity). In addition, we note that the spatial dispersion can be also approximated by a Taylor series with the first two order terms (the second term vanishes due to the symmetry of CNT). We have

(Equation 2) $$i\omega^{-1}\Pi \left(\omega ,q\right)\approx i\omega^{-1}\left[\Pi \left(\omega ,0\right)+\frac{1}{2}{\frac{\partial^{2}\Pi \left(\omega ,q\right)}{\partial q^{2}}|}_{q=0}\cdot q^{2}\right]\text{.}$$

Transforming back to position space, we perform the inverse transformation *q* → ∂/∂*z*, which suggests that the total current can be expressed as the sum of local and non-local components, i.e., *j*_*z*_ = *σ*_*zz*_(*ω*,0)*E*_*z*_ + *j*_*z*,Nonloc_, where

(Equation 3) $${j_{z,}}_{\text{Nonloc}}=-\xi \left(\omega \right)\frac{\partial^{2}E_{z}}{\partial z^{2}}\text{,}$$

with the factor of non-locality in Equation 3 given by $\xi \left(\omega \right)=\frac{1}{2}{\frac{\partial^{2}\sigma_{zz}\left(\omega ,q\right)}{\partial q^{2}}|}_{q=0}$.

Since the CNT radius is finite, the corresponding values of m remain relatively small (*m* < 60). The dominant contribution to the conductivity arises from the two intersecting bands associated with the Fermi points in different valleys of the Brillouin zone. The next step is to transform the integration over *p*_*z*_ to that over energy, and then carry it out over the range 0 < *ε* < *∞*, resulting in

(Equation 4) $$\sigma_{zz}^{\text{inter}}\left(\omega \right)=\frac{ie^{2}\hbar \omega v_{F}}{\pi^{2}R_{CN}}\int_{0}^{\infty }\frac{F\left(-\varepsilon \right)-F\left(\varepsilon \right)}{{\left[\hbar^{2}{\left(\omega +i0\right)}^{2}-4\varepsilon^{2}\right]}^{2}}\frac{d\varepsilon }{\varepsilon }\text{.}$$

The integral in Equation 4 resembles the graphene conductivity integral derived by Falkovsky et al.,^70^ however, the two expressions are not identical. The difference between the two expressions arises from the additional energy-dependent factor appearing in the denominator of the integrand. This parameter originates from the distinct dimensionality of the electronic states: in a CNT, the momentum summation reduces to an integration along two discrete one-dimensional sub-bands associated with the Fermi points, whereas in graphene it extends over the continuous two-dimensional Brillouin zone. In a similar manner, the complementary intraband conductivity can be written as

(Equation 5) $$\sigma_{zz}^{\text{intra}}\left(\omega \right)=-\frac{e^{2}v_{F}}{i\pi^{2}\hbar \omega R_{CN}}\int_{-\infty }^{\infty }\frac{\varepsilon }{\left|\varepsilon \right|}\frac{\partial F}{\partial \varepsilon }d\varepsilon \text{.}$$

The interband conductivity component dominates at high frequencies, while the intraband component is significant at low frequencies. The non-locality factor in Equation 3, can be expressed as

(Equation 6) $$\xi \left(\omega \right)=\frac{1}{2}v_{F}^{2}\frac{\partial^{2}\sigma_{zz}\left(\omega \right)}{\partial \omega^{2}}\text{,}$$

(for detailed calculations, see supplemental information^73^). The non-locality factor is a complex value, which contributes to the collision-less relaxation of a CNT too. It satisfies the Kramers-Kronig relation relation and can also be split into intraband and interband components, in a similar manner to conductivity. Our model remains accurate for *R*_*CN*_ < 30 nm, 10 nm < *L* < 1 mm, *f* < 300 THz, *μ* < 0.5 eV.

### Optical force

Let us consider the oblique incidence of a plane wave $E_{z}^{inc}\left(z\right)=-E_{0}\;\sin\;\theta_{0}e^{ikz\;\cos\;\theta_{0}}$, the optical force in CNT axially directed on a freely suspended CNT, where *θ*_0_ denotes the angle of incidence. Different formulations of the electromagnetic stress tensor yield equivalent results under appropriate conditions. For CNTs, the circle-averaged optomechanical force is evaluated by integrating the circle-averaged MST over the nanotube surface, i.e., $F=\int_{S}\overset{⃡}{T}\cdot ndS=2\pi R_{CN}\int_{-L}^{L}\left({\overset{⃡}{T}}^{\left(+\right)}-{\overset{⃡}{T}}^{\left(-\right)}\right)e_{r}dz$. Here ${\overset{⃡}{T}}^{\left(\pm \right)}$ denote the values of the stress tensor on the external and internal sides of the CNT, while **e**_*r*_ is the radial unit vector. For MST calculation, we use the electromagnetic field averaged with respect to the infinitely small element of CNT surface. As a result, any physical field is replaced by its slowly varying envelope obtained by averaging over oscillations on the scale of the crystal lattice, yielding a macroscopic field.

The macroscopic field satisfies the Maxwell equations with effective boundary conditions,^72^ which couple the electric and magnetic field components, both on the internal and external sides of CNT (for details see Supplemental Material^73^).The circle-averaged MST can also be expressed as $\overset{⃡}{T}=0.5\text{Re}\left[\left(\varepsilon_{0}EE^{*}+\mu_{0}HH^{*}\right)-0.5\cdot \left(\varepsilon_{0}{\left|E\right|}^{2}+\mu_{0}{\left|H\right|}^{2}\right)I\right]$, with **I** denoting the unit tensor. Since the CNT is slender, the field is azimuthally symmetric and has a form {**E**;**H**} = {*E*_*r*_, *E*_*z*_; *H*_*φ*_}, and thus the MST renders

$${\overset{⃡}{T}}^{\left(\pm \right)}=\frac{1}{4}\text{Re}\left(\begin{array}{ccc} T_{rr} & 0 & T_{rz}\\ 0 & T_{\varphi \varphi } & 0\\ T_{zr} & 0 & T_{zz} \end{array}\right)\text{,}$$

where

(Equation 7) $$\begin{array}{l} T_{rr}=\varepsilon_{0}{\left|E_{r}^{\left(\pm \right)}\right|}^{2}-\varepsilon_{0}{\left|E_{z}^{\left(\pm \right)}\right|}^{2}-\mu_{0}\left|H_{\varphi }^{\left(\pm \right)}\right|\\ T_{rz}=2\varepsilon_{0}E_{r}^{\left(\pm \right)}{\left(E_{z}^{\left(\pm \right)}\right)}^{*}\\ T_{\varphi \varphi }=-T_{rr}\\ T_{zr}={\left(T_{rz}\right)}^{*}\\ T_{zz}=\varepsilon_{0}{\left|E_{z}^{\left(\pm \right)}\right|}^{2}-\varepsilon_{0}{\left|E_{r}^{\left(\pm \right)}\right|}^{2}-\mu_{0}{\left|H_{\varphi }^{\left(\pm \right)}\right|}^{2} \end{array}\text{.}$$

As can be seen, the optical force acting on the CNT is directed axially and defined by the components *T*_*rz*_, *T*_*zr*_(all other MST components contribute only to CNT deformation). It is worth noting that using the local model may lead to unphysical results, since the integration of the radial component of the electric field in Equation 7 diverges due to non-integrable singularities in the diagonal elements. In contrast, when non-local effects are taken into account, these singularities are removed, and the integration in Equation 7 becomes convergent. Therefore, the inclusion of the spatial dispersion effects in the present formulation provides a physically consistent and mathematically well-posed description of optomechanical interactions in CNTs. Using the continuity of *E*_*z*_ at the CNT surface, we obtain the following expression for the longitudinal optical force $F_{z}=2\pi \varepsilon_{0}R_{CN}\cdot \text{Re}\int_{-L}^{L}\left(E_{r}^{\left(+\right)}-E_{r}^{\left(-\right)}\right)E_{z}^{*}dz$, where the discontinuity in the radial electric field is proportional to the surface charge density. By applying the continuity relation, and conducting an integration by parts, we obtain

(Equation 8) $$F_{z}=i\frac{\pi R_{CN}}{\omega }\int_{-L}^{L}\left(\frac{\partial E_{z}^{*}}{\partial z}j_{z}-\frac{\partial E_{z}}{\partial z}j_{z}^{*}\right)dz\text{.}$$

Equation 8 is the final result for the optical force, which is used in the numerical simulation of this paper.

### Integral equation

Evaluation of the force in Equation 8 requires determining the current density and electric field on the CNT surface. The integral equation approach has proven effective for solving such problems in CNT electrodynamics.^74^^,^^75^^,^^76^^,^^77^^,^^78^^,^^79^^,^^80^^,^^81^ To this end, we employ the integral equation formulation for non-local conductivity developed by Berghaus et al.^69^ The electric field in Equation 8 may be expressed through the current density and the integral equation may be solved numerically, and also permits the derivation of a simple analytical approximate solution (both approaches will be considered in this study). The integral equation has the form of a Fredholm integral equation of the second kind and reads

(Equation 9) $$j_{z}\left(z\right)-\frac{i\sigma_{zz}\left(\omega \right){\tilde{\alpha }}^{2}\left(\omega \right)}{\omega \varepsilon_{0}}\int_{-L}^{L}j_{z}\left(s\right)K\left(z,s\right)ds=B\left(z\right)\text{,}$$

with

(Equation 10) $$B\left(z\right)=\sigma_{zz}\left(\omega \right){\tilde{\alpha }}^{2}\left(\omega \right)\int_{-L}^{L}g\left(z,z^{\prime }\right)E_{z}^{inc}\left(z^{\prime }\right)dz^{\prime }\text{,}$$

where $\tilde{\alpha }\left(\omega \right)=\sqrt{\sigma_{zz}\left(\omega \right)/\xi \left(\omega \right)}$ is the special wavenumber defined by non-locality (in the local limit $\tilde{\alpha }\left(\omega \right)\to \infty$, $g\left(z,z^{\prime }\right)\to {\tilde{\alpha }}^{-2}\left(\omega \right)\delta \left(z-z^{\prime }\right)$, *δ*(*z*-*z*′) is the Dirac delta-function).

The kernel *g*(*z*, *z*′) in Equation 10, denotes the 1D Green function of the 1D Helmholtz equation satisfying the Dirichlet boundary conditions *g*(±*L*, *z*′) = 0. Accordingly, the kernel *K*(*z*, *s*) in Equation 9, is defined as

(Equation 11) $$K\left(z,s\right)=\left(\frac{\partial^{2}}{\partial s^{2}}+k^{2}\right)\int_{-L}^{L}g\left(z,z^{\prime }\right)G\left(s-z^{\prime }\right)dz^{\prime }\text{,}$$

where *G*(*s*-*z*′) = *G*(*R*_*CN*_, *s*-*z*′) and

(Equation 12) $$G\left(r,s-z\right)=R_{CN}\int_{0}^{2\pi }\frac{e^{ik\sqrt{r^{2}+R_{CN}^{2}-2rR_{CN}\;\sin\left(\phi \right)+{\left(s-z\right)}^{2}}}}{\sqrt{r^{2}+R_{CN}^{2}-2rR_{CN}\;\sin\left(\phi \right)+{\left(s-z\right)}^{2}}}d\phi \text{.}$$

Equation 12 corresponds to the 3D Green function of the 3D Helmholtz equation in a free space, which is azimuthally integrated over the CNT radius. The two kernels *g*(*z*,*z*′) and *K*(*z*,*s*) may also be written in different equivalent forms, which are most convenient for different purposes (see Supplemental Material^73^).

### Approximate analytical solution

When accounting for non-locality, one can use zero-order term in the Neumann series as an approximate solution by replacing

(Equation 13) $$j_{z}\left(z\right)=j_{z}^{\left(0\right)}\left(z\right)=-B\left(z\right)\text{,}$$

in Equation 8. This approximation corresponds to the relation $E_{z}=E_{z}^{inc}$ at the surface of CNT. After elementary integration and by virtue of Equation 8, one obtains the following result

(Equation 14) $$F_{z}=A\text{Re}\left\{\frac{{\tilde{\alpha }}^{2}\left(\omega \right)\sigma_{zz}\left(\omega \right)}{{\tilde{\alpha }}^{2}\left(\omega \right)-k^{2}\;\cos^{2}\theta_{0}}\left[L+\frac{\tilde{\alpha }\left(\omega \right)}{\sin\left(2\tilde{\alpha }\left(\omega \right)L\right)}\frac{\left(\cos\left(2\tilde{\alpha }\left(\omega \right)L\right)-\cos\left(kL\;\cos\;\theta_{0}\right)\right)}{\left({\tilde{\alpha }}^{2}\left(\omega \right)-k^{2}\;\cos^{2}\theta_{0}\right)}\right]\right\}\text{,}$$

where $A=4\pi E_{0}^{2}R_{CN}c^{-1}\;\sin^{2}\theta_{0}\;\cos\;\theta_{0}$. It demonstrates that non-locality effects qualitatively change the behavior of the optomechanical force. In the local limit $\tilde{\alpha }\left(\omega \right)\to \infty$ with fixed length the second term in Equation 14 vanishes, while the first one gives

(Equation 15) $$F_{z}=4\pi LR_{CN}E_{0}^{2}c^{-1}\;\sin^{2}\theta_{0}\;\cos\;\theta_{0}\text{Re}\left(\sigma_{zz}\left(\omega \right)\right)\text{.}$$

This limit corresponds to the homogeneous force density. The force becomes proportional to the CNT length, in agreement with Ashkin’s-like model of optical tweezing. The second term in Equation 14 is a nonmonotonic and an alternating (oscillatory) quantity with respect to the length of the CNT, a dependence which is also manifested in optical pulling. The dispersion behavior of the optical force, various in the frequency range of existence or in the absence of collision-less attenuation (ℏ*ω* > 2*μm* in the first case, and ℏ*ω* < 2*μm* in the second case). The results show that the force in CNT is “non-conservative” due to non-locality (it means that part of the provided energy is dissipated to produce mechanical action.^12^

### Optical pulling force

Using both numerical solution of the integral equation and the approximate analytical solution Equation 13, we can determine the optical pulling force acting on the CNT. For the numerical solution of the integral Equation 9, we approximate the integral in Equation 9 by a finite sum with *N* terms and select the *N* testing points at the center of each segment. As a result, we obtain a system of linear equations of *N*-th order, which was solved by matrix inversion in MATLAB.^82^

However, one can see that the kernel *G*(*z*′-*s*) is expressed in terms of a singular integral. Therefore, the change in the order of integration and differentiation is not appropriate (namely, the integral obtained in such a way is non-converging) and the resulting integral equation is ill-posed. Thus, its direct numerical solution using certain nonstandard methods, often results in some unphysical oscillations of the current density, especially near the CNT termination points. Similar oscillations are well known to exist also in the theory of classical microwave dipole antennas.^83^ A renormalization approach aimed at mitigating such numerical artifacts, was proposed by Hanson et al.^83^

Although their technique is not directly applicable to our case, due to the differences in the equation’s structure, the underlying principle remains valuable. In order to adopt such an approach for a CNT, one can use the renormalized kernel defined in the Supplemental Material.^73^ A further regularization technique is obtained by analytically integrating the singular component with the aid of the following theorem for Hankel functions (see details in Supplemental Material^73^).

Recent technological advances have enabled fabrication of CNTs with a wide range of lengths 2*L*,^64^^,^^84^ from 10 nm (ultra-short tubes) to 0.7–10 mm (exceptionally long tubes). We will consider the optical force for tubes with an experimentally achievable length within this range. Equation 16 corresponds to the threshold frequency for the onset of collisionless attenuation, which manifests as a step-like feature in the conductivity (Figure 2). Figure 3 shows the dependence of the optical force on frequency for an obliquely incident plane wave, demonstrating a good agreement between numerical simulations and the analytical solution given by Equation 13. One can see the existence of a narrow peak of the negative force, namely a pulling optical force. The frequency of the peak (maximum) is placed in the vicinity of the critical frequency satisfying the condition

(Equation 16) $$\frac{\hbar \omega }{2\mu }=1\text{.}$$

**Figure 2:**
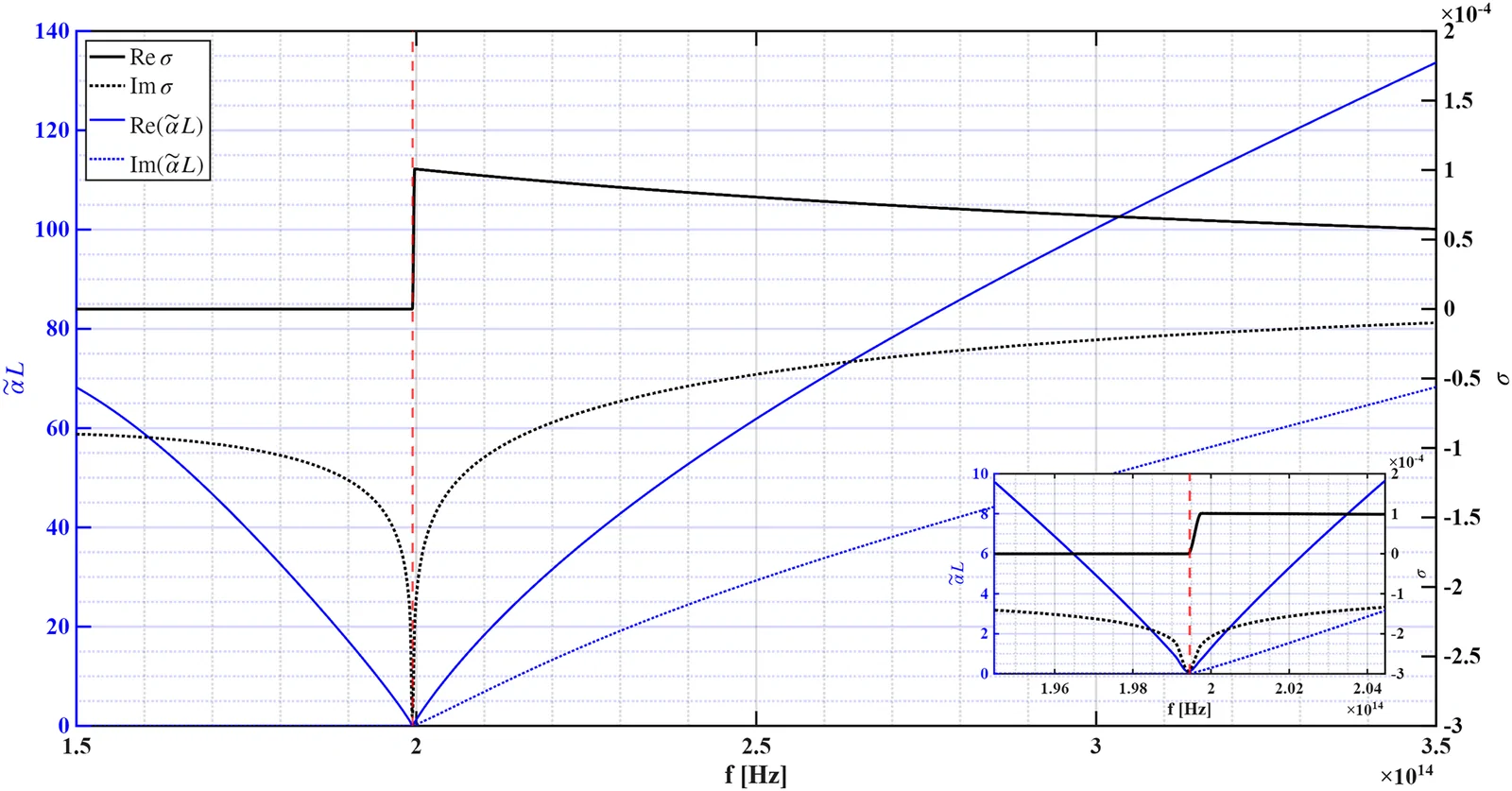
Dependency of nonlocal parameter on frequency Left axis: non-local parameter (normalized by half of the length *L* = 100 nm). Right axis: axial conductivity *σ*(*ω*) as a function of frequency for a (12,0) CNT.

**Figure 3:**
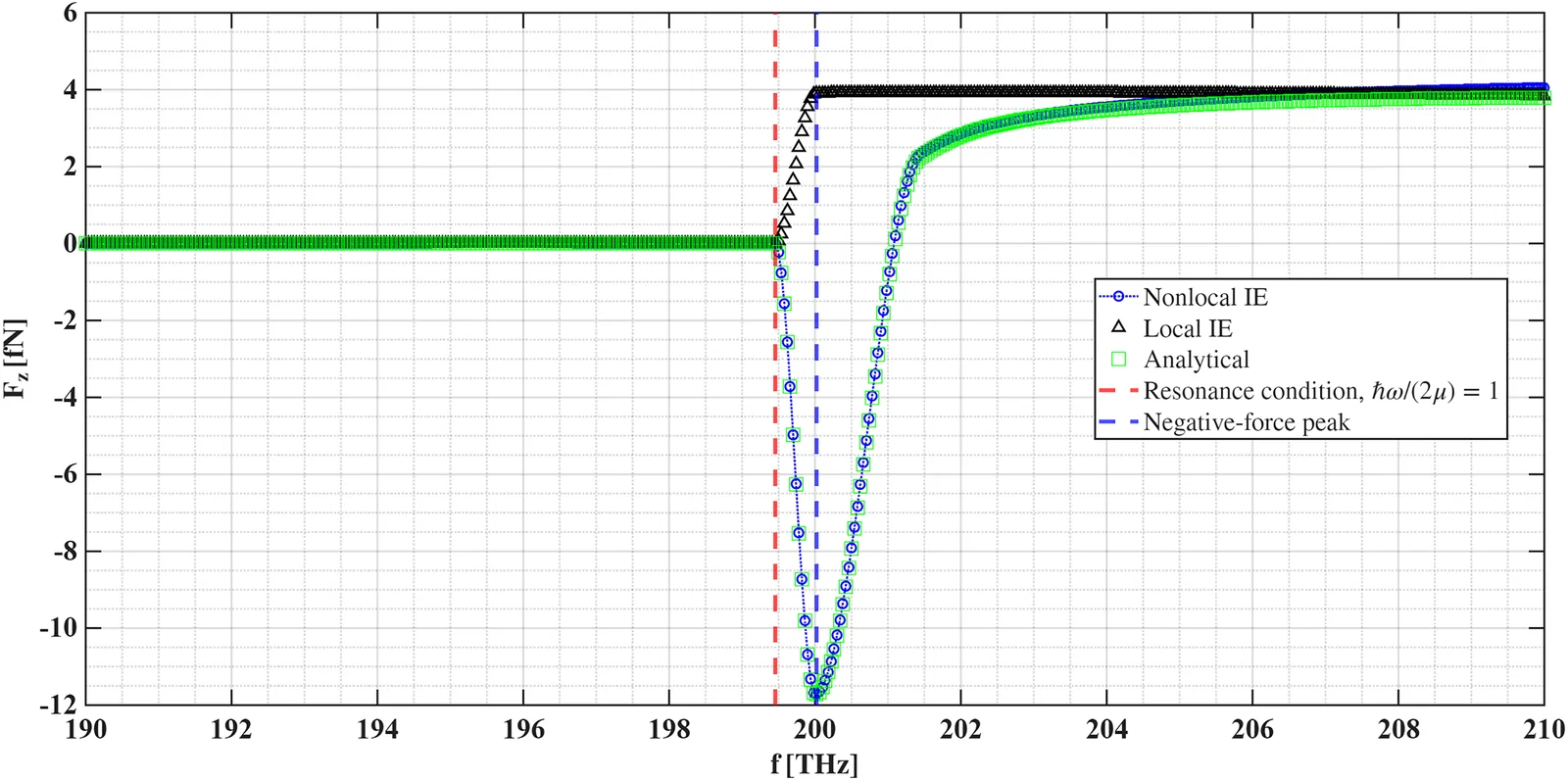
Dependence of the optical force on frequency For a (12,0) CNT corresponding to the parameters: *E*_0_ = 10^7^ V/m, *R*_*CN*_ = 0.47 nm, *μ* = 0.413eV, *L* = 100 nm, *θ*_0_ = 30°. Green squares, analytic relation; black triangles, local conductivity model; blue circles, non-local conductivity model. Number of segments for numerically solving the integral Equation 9 and computing the current density, is *N* = 411.

Figure 4 shows the dependence of the optical force on the electrochemical potential at a fixed frequency. A pronounced narrow negative peak (depending on μ) is observed, which is consistent with Equation 16. In Figure 5 the dependence of force on the CNT length (*L* is the half of the length) of a CNT for some given values of frequency and electrochemical potential. Note that we consider two principally different frequency ranges in numerical and analytical solutions. The first range corresponds to the regime (intraband transitions) defined by ℏ*ω* < 2*μm* (Figure 5A), while the second one corresponds to ℏ*ω* > 2*μm* (Figure 5B). Both figures are constructed for the local limit of $\tilde{\alpha }\left(\omega \right)\;\to \;\infty$. It is evident that the force displays a qualitatively different behavior in the two regimes, where, for example, in the first regime the force demonstrates an oscillatory behavior.

**Figure 4:**
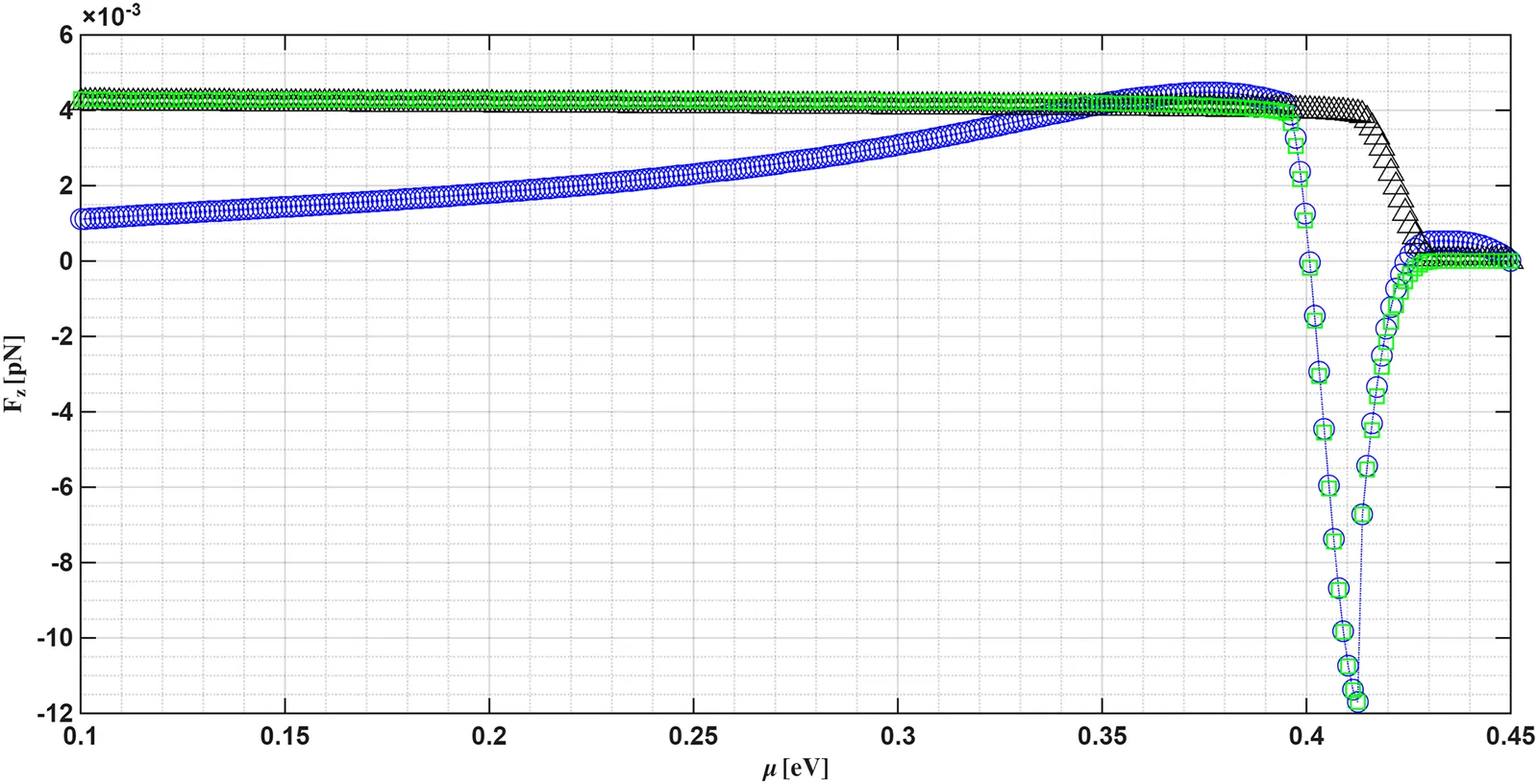
Dependence of the optical force on the electrochemical potential For a CNT (12,0): *E*_0_ = 10^7^ V/m, *R*_*CN*_ = 0.47 nm, *L* = 100 nm, *θ*_0_ = 30°, *f* = 200 THz. Green squares, analytic relation; black triangles, local conductivity model; blue circles, non-local conductivity model. The number of segments for numerically solving the integral Equation 9 and computing the current density, is *N* = 161.

**Figure 5:**
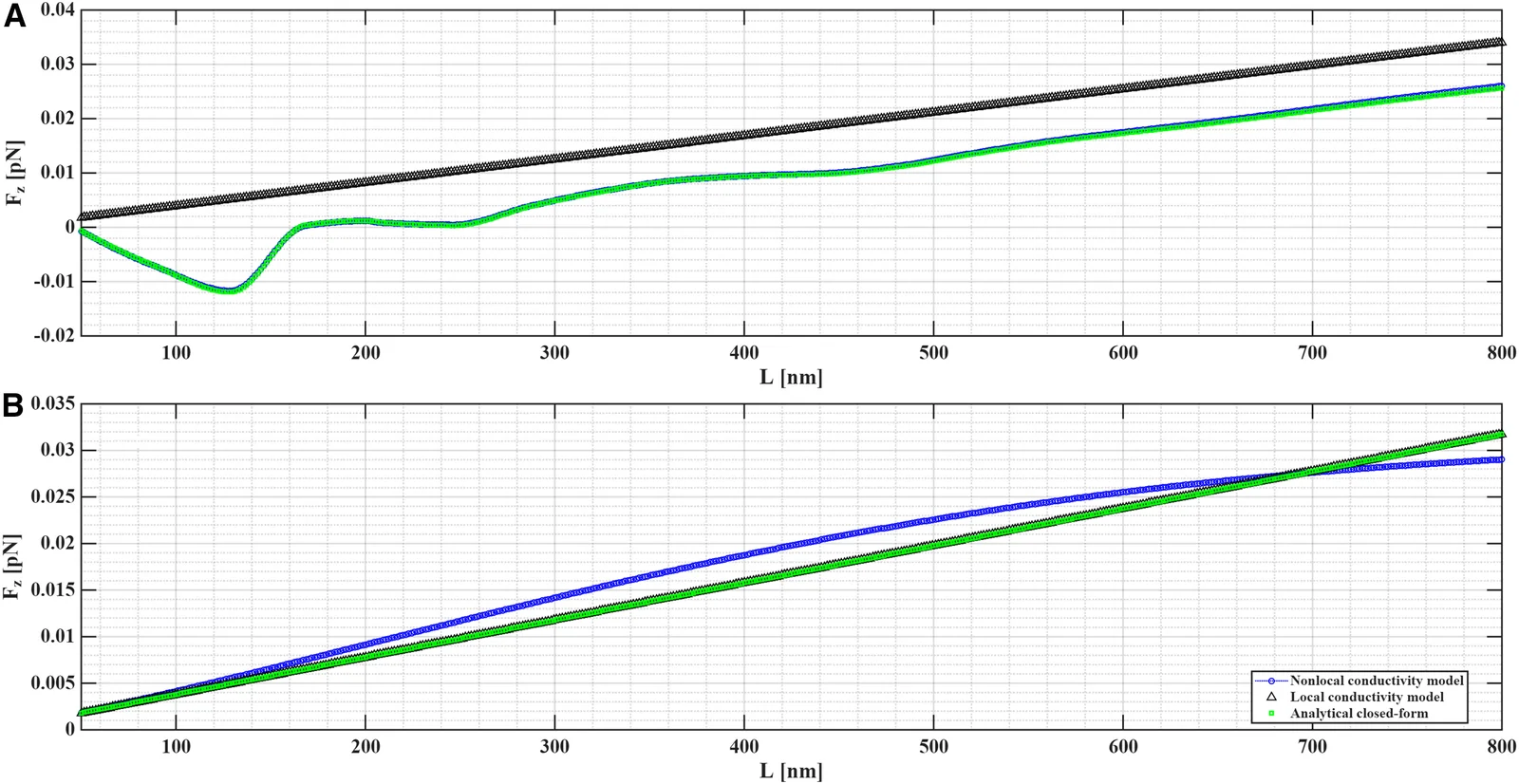
Dependence of the optical force on the length of a (12,0) CNT at two frequencies (A) *f* = 200 THz and chemical potential *μ* = 0.413*eV* and (B) *f* = 215 THz: *E*_0_ = 10^7^ V/m, *R*_*CN*_ = 0.47 nm, *θ*_0_ = 30°. Green squares, analytic relation; black triangles, local conductivity model; blue circles, non-local conductivity model. The number of segments for numerically solving the integral equation (Equation 9) and computing the current density, is *N* = 411.

For a rather short CNT the force is negative, which corresponds to the optical pulling. The numerical simulations and the analytical solution Equation 14 are in good agreement, also in determining the local limit. The computed optical force exhibits monotonic behavior and a linear dependence with respect to length. It means that the force density is uniformly distributed over the axis (the total force is given by the force density multiplied by the CNT length). Thus, we find that the effect of a pulling force is stipulated by non-locality arising solely from end effects. However, as shown, this situation is dramatically changed in the second regime. The force is positive even if non-local effects are taken into account, resulting in the common pushing (positive) force behavior. The curve obtained for the local case exactly agrees with the one which accounts for nonlocality, implying that edge effects can be ignored in this case. Note finally, that the selected cases considered in both regimes correspond to physically implementable values of CNT lengths. The calculated force magnitudes are experimentally measurable and agree with reported values in the pushing regime^85^^,^^86^ (30–50 fN for incident power 50–100 mW). Dependence of the optical force on the angle of incidence for different values of CNT length is shown on Figure 6. CNTs exhibit the strong directional behavior of optical force—rather high dependence of the force on the angle of incidence (similar to the metal nanorods^38^). It is important that pulling or pushing of the force takes place subject to the CNT length and is kept over the entire range of incidence angles considered.

**Figure 6:**
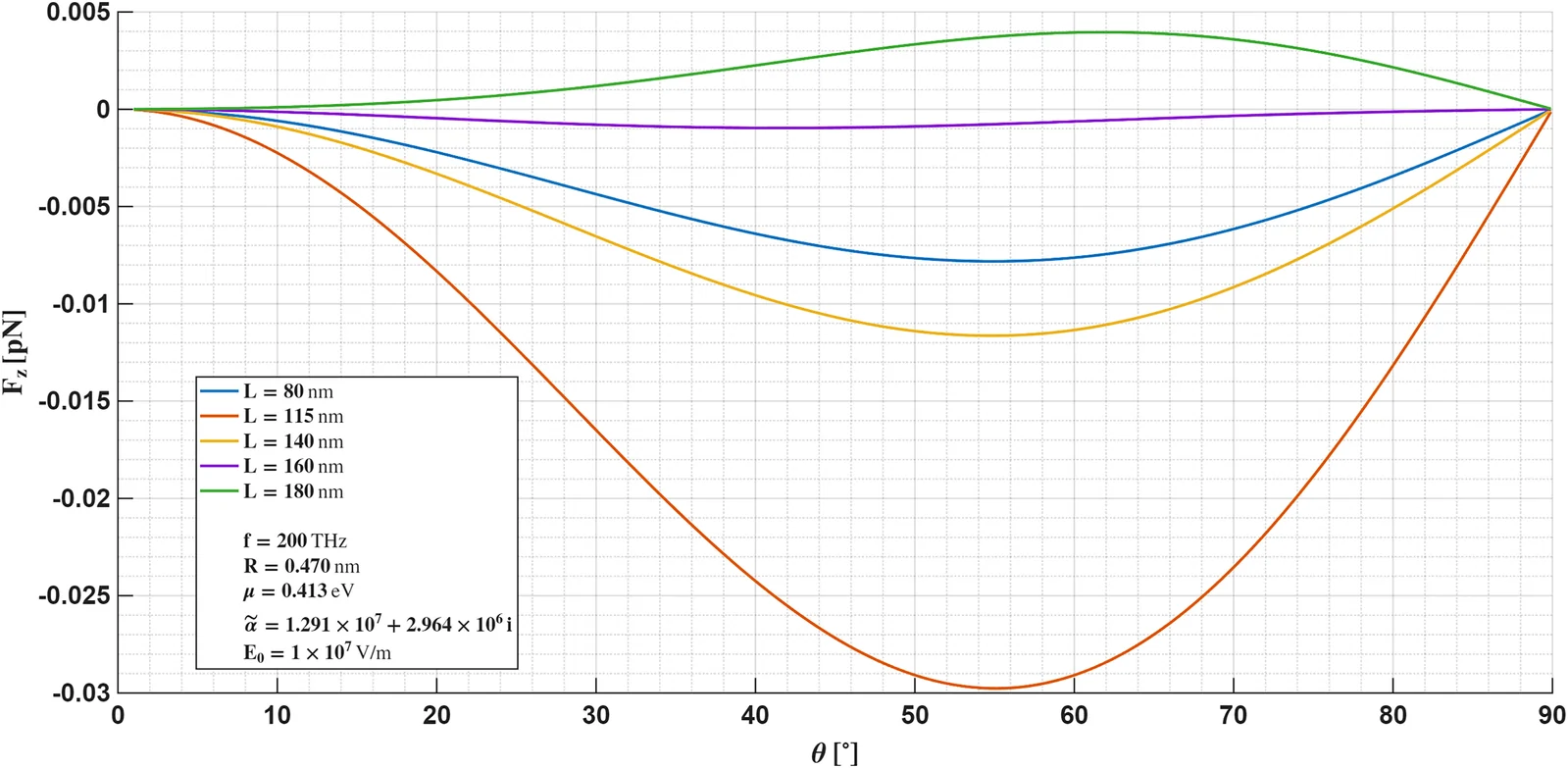
Dependence of the optical force on the angle of incidence for different CNT lengths

## Discussion

In this section we explain the pulling effect in a CNT, based on the general expression for the optical force given by Equation 8 and the approximated solution given in Equation 13. The dominant contribution to the integration presented in Equation 10, arises from the vicinity *z*′≈*z*. Therefore, we can approximate $E_{z}^{inc}\left(z^{\prime }\right)\approx E_{z}^{inc}\left(z\right)$ in Equation 10, remove it out of the integral and make elementary integration. The total field is thus given by the sum of the incident and scattering components $E\left(z\right)\approx E_{z}^{inc}\left(z\right)+E_{z}^{scatt}\left(z\right)$, where the scattering field is written as $E_{z}^{scatt}\left(z\right)\approx -E_{z}^{inc}\left(z\right)\frac{\cos\left(\tilde{\alpha }\left(\omega \right)z\right)}{\cos\left(\tilde{\alpha }\left(\omega \right)L\right)}$.

Then, by letting $E_{z}\left(z\right)\approx E_{z}^{inc}\left(z\right)$ in Equation 8, one can present the total force in the conventional form as a superposition of gradient and scattering forces,^17^^,^^18^ namely *F*=*F*_*gradient*_ + *F*_*scattering*_, where

(Equation 17) $$F_{gradient}=-\frac{\pi R_{CN}}{\omega }\text{Im}\left(\sigma_{zz}\left(\omega \right)\right)\int_{-L}^{L}\beta \left(z\right)\frac{\partial {\left|E_{z}^{inc}\right|}^{2}}{\partial z}dz\text{.}$$

(Equation 18) $$F_{scattering}=\frac{2\pi R_{CN}}{\omega }\text{Re}\left(\sigma_{zz}\left(\omega \right)\right){\left|E_{0}\right|}^{2}\int_{-L}^{L}\beta \left(z\right)\frac{\partial \Phi }{\partial z}dz\text{.}$$

The parameter *β* in the integrals is given by the relation $\beta \left(z\right)\approx 1-\frac{\cos\left(\tilde{\alpha }\left(\omega \right)z\right)}{\cos\left(\tilde{\alpha }\left(\omega \right)L\right)}$, and *E*_0_, Φ denote the amplitude force vanishes, and the scattering force can be written and phase of incident field, respectively. In the case of a plane wave the gradient as

(Equation 19) $$F_{scattering}=\frac{4\pi R_{CN}L}{\omega }\text{Re}\left(\sigma_{zz}\left(\omega \right)\right){\left|E_{0}\right|}^{2}\frac{\partial \bar{\Phi }}{\partial z}\left(1-\frac{\text{tg}\left(\tilde{\alpha }\left(\omega \right)L\right)}{\tilde{\alpha }\left(\omega \right)L}\right)\text{,}$$

where the over-line symbol means an averaging of the phase derivative with respect to the CNT axis. Since it is well known that the scattering force is defined by the losses,^17^^,^^18^ we are using the collision-less model of transport with non-collision losses that is related to the so-called Landau damping^87^ (first predicted in plasmas and thereafter found in many other systems), which is manifested by the positive term Re(*σ*_*zz*_(*ω*)) in Equation 19. However, the last factor in Equation 19 is negative in the vicinity of the resonance defined by Equation 16 due to non-locality, which is the clincher for pulling existence. The proposed physical mechanism is similar in a way to the interference of multipole field components in a dielectric sphere defining the pulling effect, as predicted in previous works.^41^ In our case, the non-locality manifests itself in the scattering end effects. As a result, the incident field induces a current in the central part of the CNT, as well as charges near the ends (these charges vanish in the local case limit). As a result, the CNT cannot be described by a simple dipole model. These induced charges at the CNT’s ends, describe the reflection mechanism of electrons and correspond to the effect of multipoles. Such additional multipoles produce a current in the direction opposite to the incident field, which in the narrow vicinity of the resonance, exceeds the induced current in the central part of the CNT due to the incident field. As a result, the current becomes opposite to the incident field, thereby producing the pulling force.

The results presented in this work are broadly applicable and enable a qualitative interpretation of Equation 14, whose accuracy is confirmed by comparison with numerical simulations. The analysis applies to achiral conducting CNTs over a wide frequency range in which the conductivity is dominated by interband transitions. Consequently, as follows from Equation 19, the scattering force is governed by the collisionless attenuation mechanism associated with Landau damping.

As expected within the framework of linear electrodynamics, the optical force is proportional to the incident light intensity. At substantially higher intensities, however, nonlinear effects arising from saturation may become significant, leading to deviations from the linear dependence. Such behavior can be described by an intensity-dependent absorption coefficient *α* given by formula *α* = *α*_0_/(1 + *I*/*I*_*Sat*_), where *I* and *I*_*Sat*_ are intensity and saturated intensity, respectively, *α*_0_ is a linear absorption coefficient.^88^ As seen from experimental data,^88^ for metallic CNTs the saturation intensity exceeds 0.1 GW/cm^2^. It strongly exceeds the value of intensity 13 MW/cm^2^, which corresponds to the incident field used in our simulations. Therefore, the linear-response approximation used here is valid.

As is seen in Figure 5B, the local and non-local results for long-length CNTs almost overlap, which is analytically clear from Equation 14. For rather high lengths, the first term becomes dominative and the force linearly dependent on the length as confirmed by the numerical results in Figure 5B. The factor before it becomes slightly dependent on parameter of non-locality, and its influence becomes negligible. It is not surprising, because as was explained before, the non-locality is governed by the end effects, whose relative contribution decreases as the CNT length increases.

In this work, we considered the simplest model of optical force in a CNT: single achiral CNT in vacuum (air) excited via a plane wave. However, the developed approach may be rather simply generalized to the more complicated structures, such as (1) CNT suspended in gas or fluid; (2) appearance of different environments, for example, dielectric, metallic, and plasma backgrounds, or adding one more CNT; (3) CNT placing in the waveguide or optical cavity. Such generalizations are promising for enhancing the of nanostructures force, as has been demonstrated for other types (see, for example Li et al.^40^). The modification of CNT molecular structure opens the ways for different applications of CNT manipulation. Among them are: (1) using the pulling (Figure 4) for measurement of CNT electrochemical potential after substitutional doping of CNT^89^; using the optical pulling force for CNT mechanical sensing^90^^,^^91^; applications of the optical force for CNT sorting^92^ with respect to the length or radius.

In summary, we have developed a consistent theory for optical forces in CNTs with non-local conductivity (spatial dispersion). Unlike most contributions which consider 1D CNT (infinite length), it is demonstrated here that a rigorous account of CNT edge effects (finite length) can dramatically affect its optomechanical properties. The present formulation is based on implying a Fredholm integral equation of the second kind for the axial component of the surface current. The optical force is simulated by the numerical solution of an integral equation as well as by an approximate analytical solution. An excellent agreement is found between these two approaches. We demonstrate for the first time that there exists a negative optical force in a CNT (optical pulling). The pulling effect is stipulated by the non-locality of the conductivity, and it disappears in the local limit. The occurrence of optical pulling is typical for relatively short CNTs (100 nm–200 nm). For larger values of length, the influence of spatial dispersion decreases and ceases to exhibit itself at large lengths. The force becomes positive (pushing) and linearly depends on the length. Yet another possible way to obtain an optical pulling force in a CNT is by increasing the forward scattering by means of a structured light, such as a Gaussian beam, Bessel beam, as well as other tractor beams. It is expected that including spatial dispersion considerations of constitutive parameters, may be able to produce optical forces pulling also in other media. Our work paves a way for future studies of optical forces in nanostructures.

### Limitations of the study

The main limitation of the developed theory of optical forces in a CNT with non-local conductivity is the case of achiral CNT. Among other important limitations are open-end single-wall CNTs with metallic conductivity. The end configuration changes the behavior of the current near the ends and, correspondently. the kernel of integral equation. As a result, the approximate solution for the force should be modified too. In addition, the influence of saturation on the optical force in CNT is only simply estimated, while the detailed analysis of non-linear optical effects on tweezing of CNTs is not given for brevity. Future research activity on CNT pulling-force, will be mainly focused on determining the influence of chirality and ends configuration. Any experimental validation of the newly predicted effects reported in this study is certainly welcome and it will open the way for novel applications in optical tweezing.

## Resource availability

### Lead contact

Further information and requests should be directed to and will be fulfilled by the lead contact, Gregory Ya. Slepyan (gregory_slepyan@yahoo.com).

### Materials availability

This study did not generate new unique reagents.

### Data and code availability

• Data: The data supporting the findings of this study have been deposited in Zenodo and are publicly available at https://doi.org/10.5281/zenodo.22696791.
• Code: The MATLAB code used to generate the results reported in this paper has been deposited in Zenodo and is publicly available at https://doi.org/10.5281/zenodo.22085841.
• Other items: Any additional information required to reanalyze the data reported in this paper is available from the lead contact upon request.

## Acknowledgments

T.B. and T.M. acknowledge the partial support of 10.13039/501100003977ISF grant no. 3858/24.

## Author contributions

Conceptualization, T.B., T.M., and G.Ya.S.; investigation, T.B., T.M., and G.Ya.S.; writing – original draft and review and editing, T.B., T.M., and G.Ya.S.

## Declaration of interests

The authors declare no competing interests.

## Declaration of generative AI and AI-assisted technologies in the writing process

During the preparation of this manuscript, the authors used generative AI tools to assist with improving the language, grammar checking, code readability, and clarity of the text, all originally written by the authors. After using this tool, the authors reviewed and edited the content as needed and take full responsibility for the content of the publication.

## STAR★Methods

### Key resources table

**Table:** 

REAGENT or RESOURCE	SOURCE	IDENTIFIER
**Deposited data**		

Numerical/source data underlying Figure S1, 3–5	This paper	https://doi.org/10.5281/zenodo.22696791

**Software and algorithms**		

MATLAB R2025b Update 5	MathWorks	https://www.mathworks.com/products/matlab.html
Original MATLAB code used to solve the Fredholm integral equation and calculate the optical force	This paper	https://doi.org/10.5281/zenodo.22085841

### Method details

#### Linear response theory

We analyze the transport properties of finite-length CNTs based on the concept of generalized susceptibility, which determines the response of charge carriers in a CNT to a perturbation, with spatial and temporal variations. For this purpose, we apply the general Kubo formula for the CNT conductivity, which couples the current density (a response) to the applied electromagnetic field. The applicability of using a linear response theory is clearly restricted to the regime where the current density changes linearly with the field. In our model, the field perturbation at a given point depends not only on the force at the same point, but also on its vicinity (nonlocal response). As a result, the CNT conductivity is presented in the form of a linear differential operator (Equation 3). This special form is then used for formulating the associated electrodynamic boundary-value problem, and eventually to evaluating the optical forces. A detailed derivation of the nonlocal conductivity model is provided in Methods S1.

#### Method of effective boundary conditions

This method is used for the independent formulation and solution of the boundary-value problem for a finite-length CNT. For this purpose, we employ the concept of a macroscopic electromagnetic field (field averaged with respect to the infinitely small volume). From another point of view, one can also consider an envelope of an oscillating field with respect to the hexagonal carbon crystal lattice, which is rolled onto the CNT cylindrical radius. This averaging procedure is made with respect to a 2D surface, in contrast to 3D lattice employed in different conventional cases.^54^ As a result, we obtain a practical useful simplification for the resulting boundary-value problem governed by the Maxwell equations, by applying a special canonical form of effective boundary conditions, in the form of two-side impedance boundary conditions. The impedance is determined from the Kubo formula by using a linear response theory.

#### Method of integral equations

The electromagnetic field in the whole space (both inside and outside the CNT) is induced by the current distributed on the CNT surface. The scattered electromagnetic field is presented in the form of an integral over the CNT surface. Using effective boundary conditions, we obtain a Fredholm integral equation of the second type for the current density. For the purpose of obtaining a numerical solution, we replace the integral by using a quadrature discretized scheme variables. The kernel of the integral equation has a weak (integrable) singularity. Two different equivalent forms for the kernel are obtained (details are presented in the supplemental material). After approximating the integral and using discretization, we obtain a linear algebraic system, which can be inverted using standard methods of linear algebra. In the course of the numerical simulations, we have used the independent two forms of the kernels in order to check the accuracy of the numerical scheme. An exact agreement was obtained between the two cases. The integral equation is derived in Methods S2, and its two equivalent kernel representations are derived in Methods S3.

#### Numerical implementation

For the numerical solution of the Fredholm integral equation (Equation 9), the CNT axial domain ([-*L, L*]) was divided into (*N*) segments, and the testing points were selected at the centers of the corresponding segments. The integral was approximated by a finite sum, resulting in an (*N*)-th-order system of linear algebraic equations for the axial surface-current density. The singular behavior of the integral-equation kernel was treated using the regularized kernel formulation described above. Two equivalent forms of the kernel were independently implemented and compared to verify the accuracy of the numerical procedure. The number of segments (*N*) used for each calculation is specified in the corresponding figure legend. The resulting system was solved numerically using MATLAB.^82^ The code used to perform the numerical calculations and generate the results presented in this study is publicly available on https://doi.org/10.5281/zenodo.22085841. A numerical comparison of the two equivalent kernel representations is presented in Figure S1.

#### Simulation parameters

Numerical calculations were performed for a metallic zigzag (12,0) CNT with radius *R*_*CN*_ = 0.47 nm and incident electric-field amplitude *E*_0_ = 10^7^ V/m. Unless otherwise specified, the incident plane wave was applied at an angle *θ*_0_ = 30° with respect to the CNT longitudinal axis. The electrochemical potential *μ*, CNT half-length *L*, excitation frequency *f*, and incidence angle *θ*_0_ were varied according to the parameter study considered. For the frequency-dependent calculations of the nonlocality parameter and axial conductivity (Figure 2), a (12,0) CNT with *L* = 100 nm was considered. For the frequency dependence of the optical force (Figure 3), the parameters were *R*_*CN*_ = 0.47 nm, *μ* = 0.413 eV, *L* = 100 nm, *θ*_0_ = 30°, and *E*_0_ = 10^7^ V/m; the integral equation was discretized using *N* = 411 segments. For the electrochemical-potential dependence of the optical force (Figure 4), the parameters, were *R*_*CN*_ = 0.47 nm, *L* = 100 nm, *f* = 200 THz, *θ*_0_ = 30°, and *E*_0_ = 10^7^ V/m, with *N* = 161.For the CNT-length dependence (Figure 5), the half-length *L* was varied while, *R*_*CN*_ = 0.47 nm *θ*_0_ = 30°, and *E*_0_ = 10^7^ V/m were fixed. Two frequency regimes were considered with *μ* = 0.413 eV: *f* = 200 THz and *f* = 215 THz. The numerical integral equation was solved using *N* = 411 segments. For the angular dependence of the optical force (Figure 6), the incidence angle *θ*_0_ was varied at *R*_*CN*_ = 0.47 nm, *E*_0_ = 10^7^ V/m, *μ* = 0.413 eV, and *f* = 200 THz for several CNT half-lengths *L*.

#### Calculation of optical forces in CNT

The proposed theory of optical force is based on the Maxwell stress tensor (MST) formulation. The total optical force is presented in the form of the integral of MST over the two-sided CNT surface. Two different approaches are used for the MST calculations. The first is based on using a direct numerical solution of the integral equation and in the second one, we employ an approximation based on guessing of an initial solution of a Neumann series. Finally, we are able to obtain a rather simple analytical relation for the optical force, which precisely coincides with the numerical simulations and is thus supported to a high degree of accuracy. The newly obtained solution allowed us to explicitly predict and physically explain the effect of optical pulling in CNT, which is the main result of this work.

### Quantification and statistical analysis

This study is theoretical and computational and does not involve experimental samples, pooled data, or statistical hypothesis testing. The reported results were obtained through deterministic numerical solutions of the integral equation and analytical calculations. Numerical computations were performed using MATLAB R2025b Update 5. Numerical accuracy was assessed by comparing two equivalent kernel formulations and by comparing the numerical results with the approximate analytical solution.
