# Supplementary material for: Optical pulling force in carbon nanotubes: Manifestation of non-local conductivity
Source: iScience. 2026 Oct 13;29(11):117817. doi: 10.1016/j.isci.2026.117817 (PMC13683856; doi:10.1016/j.isci.2026.117817)
Supplement: Supplementary file 1 [file mmc1.pdf]

iScience, Volume *29*

Supplemental information

Optical pulling force in carbon nanotubes: Manifestation of non-local conductivity

Tomer Berghaus, Touvia Miloh, and Gregory Ya. Slepyan

Supplementary Materials

This document provides supplementary derivations supporting the theoretical formulation presented in the main text. Methods S1 derives the local and nonlocal components of the axial CNT conductivity used in Equations 1–6. Methods S2 derives the Fredholm integral equation for the axial surface-current density given in Equation 9. Methods S3 derives two equivalent regularized representations of the integral-equation kernel used in the numerical calculations.

METHODS S1: NONLOCAL CONDUCTIVITY MODEL, RELATED TO EQUATIONS 1–6.

We derive the CNT conductivity using the Dirac-pseudospin model [S1]. This model applies from the THz to the visible-frequency range and accounts for interband transitions. The corresponding Hamiltonian is $\hat{H} = \hat{H}_K + \hat{V}(t)$, where $\hat{H}_K = -\hbar v_F \hat{\sigma}_z \frac{\partial}{\partial z}$ represents the Hamiltonian of the conduction electrons in the absence of the electromagnetic field, ($\hbar$ is the reduced Planck constant, v_F denotes the Fermi velocity and $\hat{\sigma}_z = (0,-i;i,0)$ is the longitudinal Pauli matrix). In addition, we define $\hat{V}(t) = -eE_z\hat{z}$ (*e* denotes the electron charge, E_z is the longitudinal component of electric field and $\hat{z}$ is the operator of longitudinal coordinate). We solve the Liouville equation for the 2×2 pseudospin matrix $i\hbar\frac{\partial\hat{\rho}}{\partial t} = \left[\hat{H}_K, \hat{\rho}\right] + \left[\hat{V}(t), \hat{\rho}\right]$, treating $\hat{V}(t)$ as a small perturbation. The formulation is based on assuming a monochromatic EM-field, which means letting $\hat{V}(t) = \mathrm{Re}\left(\hat{V}e^{-i\omega t}\right)$ and $\hat{\rho}(t) = \hat{\rho}_0 + \mathrm{Re}\left(\delta\hat{\rho}\cdot e^{-i\omega t}\right)$, where $\delta\hat{\rho}$ represents a small perturbation to the equilibrium density matrix. In addition, $\hat{\rho}_0 = F(\varepsilon) = \left[1+\exp(\varepsilon-\mu)/k_BT\right]^{-1}$ denotes the equilibrium Fermi–Dirac distribution, ε is the electron's energy, μ is the electrochemical potential, k_B represents the Boltzmann constant, and *T* is the temperature.

The electromagnetic fields and induced currents can be expressed as superpositions of traveling waves of the form $\exp\left[i\left(qz-\omega t\right)\right]$, which propagate in opposite directions (different signs of wavenumbers *q*). We restrict the analysis to the axisymmetric response, corresponding to an axial current induced by the axial electric field. All other components of the 2×2 conductivity tensor, are assumed to be zero. Far from the CNT terminations (ends), their interaction can be accurately modeled by means of using the corresponding infinite-length CNT approximation. Reflections from the extreme ends of the CNT and accounting for nonlocal effects, are also incorporated by applying the additional boundary conditions [S2]. Following the Kubo technique, the perturbation in the elements of the density matrix, expressed in the momentum space, can be represented as

$$\left(\delta\hat{\rho}\right)_{mn,p,p+q} = \frac{F\left(\varepsilon_{m,p+q}\right) - F\left(\varepsilon_{np}\right)}{\left[\varepsilon_{np} - \varepsilon_{m,p+q} + \hbar\omega + i0\right]}\hat{V}_{mn,p,p+q} \tag{S1}$$

where $\varepsilon_{m,p}$ is the energy of the electron with momentum p in the sub-band indexed by m. The conductivity in the momentum space reads

$$\sigma_{zz}(\omega,q)=\frac{i}{\omega}\left[\Pi(\omega,q)-\Pi(0,q)\right] \tag{S2}$$

where

$$\Pi(\omega,q)=\sum_{mn}\sum_{p}\frac{F(\varepsilon_{m,p+q})-F(\varepsilon_{np})}{\left[\varepsilon_{np}-\varepsilon_{m,p+q}-\hbar(\omega+i0)\right]}\left|\langle\hat{j}_z\rangle_{mn,p,p+q}\right|^2 \tag{S3}$$

is defined as the polarizability and $\langle\hat{j}_z\rangle_{mn,p,p+q}$ represent the matrix elements of the operators of the current density. The sum over p in Equation (S3) accounts for states with different momenta and represents the conventional integration over the Brillouin zone. It is also convenient to decompose the total nonlocal conductivity into interband and intraband components, namely, $\sigma_{zz}(\omega,q)=\sigma_{zz}^{\text{inter}}(\omega,q)+\sigma_{zz}^{\text{intra}}(\omega,q)$. The terms with $m\neq n$ in Equation (S3) correspond to interband transitions while the other terms correspond to intraband transitions (which constitute the Drude contribution).

To facilitate the incorporation of the nonlocal effects, we employ a Taylor series expansion of Equation (S3) with respect to the momentum *q*, by retaining the second-order approximation (Equation 2 of the main text). We will adopt the approximation $\langle\hat{j}_z\rangle_{mn,p,p+q}\approx\langle\hat{j}_z\rangle_{mn,pp}$ along with the following dispersion relation for the Dirac fermi $\varepsilon_{mp}\approx\pm v_F p$. Since the current in the CNT is supported by contributions from all available states, (S3) can be recast as

$$\Pi(\omega,q)=\sum_{mn}\sum_{p}F(\varepsilon_{np})\left|\langle\hat{j}_z\rangle_{mn,pp}\right|^2\left\{\frac{1}{\left[\varepsilon_{np}-\varepsilon_{m,p+q}-\hbar(\omega+i0)\right]}+\frac{1}{\left[\varepsilon_{np}-\varepsilon_{m,p+q}+\hbar(\omega+i0)\right]}\right\} \tag{S4}$$

Returning to the position space, we perform an inversion $q\to\partial/\partial z$, which suggests that the total current can be expressed as the sum of local and nonlocal components, i.e., $j_z=\sigma_{zz}(\omega,0)E_z+j_{z,\text{Nonloc}}$

where

$$j_{z,\text{Nonloc}}=-\xi(\omega)\frac{\partial^2 E_z}{\partial z^2} \tag{S5}$$

with the factor of nonlocality defined as

$$\xi(\omega)=\frac{1}{2}\frac{\partial^2\sigma_{zz}(\omega,q)}{\partial q^2}\bigg|_{q=0} \tag{S6}$$

and for brevity we define $\sigma_{zz}(\omega,0)=\sigma_{zz}(\omega)$. Similarly, the nonlocal current can be decomposed into interband and intraband contributions, yielding $\xi(\omega)=\xi^{\text{inter}}(\omega)+\xi^{\text{intra}}(\omega)$. Combining Equations (S2), (S4) and (S5) finally renders

$$\xi(\omega)=4i\hbar(\hbar v_F)^2\sum_{mn}\sum_{p}\frac{\left(F(\varepsilon_{np})-F(\varepsilon_{mp})\right)\left|\langle\hat{j}_z\rangle_{mn,pp}\right|^2}{\left[\varepsilon_{np}-\varepsilon_{mp}+\hbar(\omega+i0)\right]^3(\varepsilon_{np}-\varepsilon_{mp})}\,. \tag{S7}$$

Nonlocality is evident in (S5) through the presence of the second-order spatial derivative, indicating that the current density at a given point depends not only on the value of the electric field at that point, but also on its spatial vicinity. Note that the term corresponding to the first-order derivative in the Taylor series vanishes owing to axial symmetry of achiral CNTs, which ensures invariance under rotation around the tube axis. Taking into account that $\partial^2\left(x^{-1}\right)/\partial x^2 = 2x^{-3}$, we obtain

$$\frac{2\hbar^2}{\left[\varepsilon_{np} - \varepsilon_{m,p+q} \pm \hbar\omega\right]^3} = \frac{\partial^2}{\partial\omega^2}\left(\frac{1}{\varepsilon_{np} - \varepsilon_{m,p+q} \pm \hbar\omega}\right) \tag{S8}$$

and by virtue of (S8), we can rewrite (S7) in the form:

$$\xi\left(\omega\right) = -2i\hbar v_F^2 \frac{\partial^2}{\partial\omega^2}\sum_{mn}\sum_{p}\frac{\left(F\left(\varepsilon_{np}\right) - F\left(\varepsilon_{mp}\right)\right)\left|\left\langle \hat{j}_z\right\rangle_{mn,pp}\right|^2}{\left[\varepsilon_{np} - \varepsilon_{mp} + \hbar\left(\omega + i0\right)\right]\left(\varepsilon_{np} - \varepsilon_{mp}\right)} \tag{S9}$$

which immediately yields Equation (6) of the main text.

In order to simplify the general Kubo formalism for low-energy excitations constrained by the condition $E < 1$ eV, we adapt it to the case of a pseudo-spin electron liquid, in a similar manner to the approach used for a monolayer graphene in [S3]. To this end, we consider the electronic states near the K-points and employ a linear approximation for $\varepsilon_{\mathbf{p}} = \pm v_F\left|\mathbf{p} - \mathbf{p}_F\right|$. We will also consider the case of low temperature ($T << \mu$) and a collision-free electron motion ($\nu = 0$). In contrast with graphene[S1], [S3], for a CNT we take into account the transverse electron confinement (the azimuthal component of electron momentum is discrete, while the longitudinal component is continuous).

Following [S4], we refine the Kubo formalism to suit the specific model under consideration. For a zigzag CNT (*m*, 0), we have

$$\sigma_{zz}^{\text{inter}}\left(\omega\right) = \frac{ie^2\omega}{2\pi^2\hbar R_{CN}}\sum_{s=1}^{m}\int_{BZ}\frac{\left|v_{cv}\left(p_z,s\right)\right|^2}{\varepsilon\left(p_z,s\right)}\cdot\frac{F\left[-\varepsilon\left(p_z,s\right)\right] - F\left[\varepsilon\left(p_z,s\right)\right]}{\hbar^2\left(\omega + i0\right)^2 - 4\varepsilon^2\left(p_z,s\right)}dp_z \tag{S10}$$

where $v_{cv}\left(p_z,s\right)$ denotes a velocity matrix element of direct interband transitions [S4]. For future considerations, it may be simply approximated by[4] $v_{cv}\left(p_z,s\right) \approx \hbar v_F$ [64].

Real implemented CNTs have a finite radius, which corresponds to rather small values of *m* (*m*<60). The main contribution to the conductivity is provided by the Fermi points *s*=*m*/3 and *s*=2*m*/3, which correspond to the different valleys of the BZ zone. Next, we transform the integration over p_z to that over energy and carry it out over the range $0 < \varepsilon < \infty$, which results in Equations paper (4) and (5) in our paper. For the case where $\mu >> k_B T$ (named zero-temperature approximation), the final result reads

$$\sigma_{zz}^{\text{inter}}(\omega)=\frac{e^2 v_F}{2\pi\hbar\omega}\left(H(\hbar\omega-2\mu)-\frac{i}{\pi}\ln\left|\frac{4\mu^2}{4\mu^2-(\hbar\omega)^2}\right|\right) \tag{S11}$$

where $H(x)$ is the Heaviside step function. The conductivity (S11) goes to infinity in the limit $\mu\to 0$, in accordance with the zero-temperature approximation. The nonlocality parameter ξ, can be written as

$$\xi^{\text{inter}}(\omega)=i\frac{16\sigma_0\mu v_F^2}{\pi\hbar\omega^3}\left\{1-\frac{(\hbar\omega)^4}{\left[(\hbar\omega)^2-4\mu^2\right]^2}\right\} \tag{S12}$$

where σ_0 denotes the quantum of conductivity. The singularity in (S11), (S12) at the step point $\hbar\omega-2\mu$, is regularized by finite-temperature effects [S2]. The applied longitudinal electric field also induces transitions between electronic states within the same partially occupied subband, producing the intraband response. Within the low-energy approximation adopted above, the corresponding longitudinal conductivity is given by

$$\sigma_{zz}^{\text{intra}}(\omega)=-\frac{2ie^2 v_F}{\pi^2\hbar\omega R_{CN}}\tanh\left(\frac{\mu}{2k_B T}\right) \tag{S13}$$

METHODS S2: DERIVATION OF THE INTEGRAL EQUATION, RELATED TO EQUATIONS 9–12.

The field scattered by the CNT can be described by the longitudinal component of the electric Hertz vector Π, as

$$E_z^{sc}=\frac{\partial^2\Pi}{\partial z^2}+k^2\Pi\ . \tag{S14}$$

This field is governed by the three-dimensional wave equation derived from Maxwell's equations, which can be expressed in terms of the electric Hertz vector as

$$\left(\nabla^2+k^2\right)\Pi=-\frac{i}{\omega\varepsilon_0}\delta\left(r-R_{CN}\right)j_z \tag{S15}$$

where $\delta(x)$ is the Dirac delta function. The Hertz potential satisfies the effective boundary conditions imposed on the CNT surface. The discontinuity in the tangential component of the magnetic field is proportional to the surface current density, while the tangential component of the electric field remains continuous across the surface. Denoting the interior $(R_{CN}-0)$ and the exterior $(R_{CN}+0)$ components of the Hertz vector by $\Pi^{(\pm)}=\Pi\left(r=R_{CN}\pm 0\right)$, the effective boundary conditions for the eigenmodes are given by

$$\Pi^{(+)}-\Pi^{(-)}=0,\quad -\infty<z<\infty \tag{S16}$$

$$\left(1+\frac{1}{\tilde{\alpha}^2(\omega)}\frac{\partial^2}{\partial z^2}\right)\frac{\partial}{\partial r}\left(\Pi^{(+)}-\Pi^{(-)}\right)=\frac{\sigma_{zz}(\omega)}{i\omega\varepsilon_0}\left(\frac{\partial^2}{\partial z^2}+k^2\right)\Pi+\frac{\sigma_{zz}(\omega)}{i\omega\varepsilon_0}E_z^{inc}, \quad -L<z<L \tag{S17}$$

$$\frac{\partial}{\partial r}\left(\Pi^{(+)}-\Pi^{(-)}\right)=0, \quad |z|>L \tag{S18}$$

where the subscript $\pm$ in the right-hand part of (S17) is omitted owing to continuity. The solution of the inhomogeneous Helmholtz equation (S15) for the Hertz potential can be expressed as

$$j_z(z)=-\sigma_{zz}(\omega)\tilde{\alpha}^2(\omega)\int_{-L}^{L}g(z,z')E_z(z')dz' \tag{S19}$$

where $g(z,z')$ represents the corresponding Green function of Sturm-Liouville equation

$$\frac{\partial^2 g(z,z')}{\partial z^2}+\tilde{\alpha}^2(\omega)g(z,z')=\delta(z-z') \tag{S20}$$

with a Dirichlet-type boundary conditions $g(\pm L,z')=0$. These boundary conditions guarantee satisfying the so-called 'additional' boundary conditions[2] and are necessary for the consistent (correct) description of nonlocality[2].

The Green function may be presented in the following form,

$$g(z,z')=-\frac{1}{\tilde{\alpha}(\omega)\sin\left(2\tilde{\alpha}(\omega)L\right)}\Big[H(z'-z)\sin\left(\tilde{\alpha}(\omega)(z+L)\right)\sin\left(\tilde{\alpha}(\omega)(z'-L)\right)+ \tag{S21}$$
$$H(z-z')\sin\left(\tilde{\alpha}(\omega)(z'+L)\right)\sin\left(\tilde{\alpha}(\omega)(z-L)\right)\Big]$$

where $H(z)$ is the Heaviside function. Another form, which is based on using eigenmodes, reads

$$g(z,z')=\sum_{n=1}^{\infty}\frac{u_n(z)u_n(z')}{\left(\frac{n\pi}{2L}\right)^2-\tilde{\alpha}^2(\omega)} \tag{S22}$$

where $u_n(z)=\sqrt{\frac{2}{L}}\sin\left(\frac{n\pi}{2L}(z+L)\right)$. In the local limit $|\tilde{\alpha}(\omega)|\to\infty$, Equation (S21) renders

$g(z,z')=-\tilde{\alpha}^{-2}(\omega)\delta(z-z')$.

Let us next express the Hertz potential as

$$\Pi(r,z)=\frac{i}{\omega\varepsilon_0}\int_{-L}^{L}j_z(s)G(r,z-s)ds \tag{S23}$$

where $j_z(z)$ represents the unknown current density and the kernel is given by

$$G(r,s-z)=R_{CN}\int_0^{2\pi}\frac{e^{ik\sqrt{r^2+R_{CN}^2-2rR_{CN}\sin(\phi)+(s-z)^2}}}{\sqrt{r^2+R_{CN}^2-2rR_{CN}\sin(\phi)+(s-z)^2}}d\phi\ . \tag{S24}$$

Substituting Equation (S19) into Equation (S20), yields the exact solution to Maxwell's equations throughout the entire space, by satisfying both the radiation condition and the boundary conditions expressed in Equation (S17). The surface current density appearing in Equation (S23) must then be determined by enforcing the boundary condition (S18). After substitution of Equation (S23) into Equation (S18), we obtain the governing equation for the surface-current density

$$j_z(z)+\frac{i\sigma_{zz}(\omega)\tilde{\alpha}^2(\omega)}{\omega\varepsilon_0}\int_{-L}^{L}g(z,z')\left(\frac{\partial^2}{\partial z'^2}+k^2\right)\left\{\int_{-L}^{L}j_z(s)G(s-z')ds\right\}dz'=B(z) \tag{S25}$$

where $G(s-z')=G(R_{CN},s-z')$ is given by Equation (S24) and

$$B(z)=\sigma_{zz}(\omega)\tilde{\alpha}^2(\omega)\int_{-L}^{L}g(z,z')E_z^{inc}(z')dz'\ . \tag{S26}$$

Introducing the kernel

$$K(z,s)=\left(\frac{\partial^2}{\partial s^2}+k^2\right)\int_{-L}^{L}g(z,z')G(s-z')dz' \tag{S27}$$

and accounting for (S26), we finally obtain the integral equation (9).

METHODS S3: EQUIVALENT FORMS OF THE KERNEL OF INTEGRAL EQUATION 9.

Representation based on singular integrals

A singular-integral transformation was developed in [S5] in the context of wire-dipole antennas. However, the integral forms for wires and CNTs are not identical because of the boundary conditions. Therefore, we derive the corresponding transformation explicitly. Let us consider the configuration of the CNT depicted at Fig.1. The symbol Σ denotes the surface of the CNT ($d\Sigma=2\pi R_{CN}dzd\phi$ is the differential of this surface). The symbol Σ_δ denotes an excluded surface element introduced to isolate the singularity during the integral transformation. The symbols C,C_δ represent the two-side closed rims of the CNT and the excluded element, respectively. Next we consider the integral

$$\nu(z')=\lim_{\delta\to 0}\int_{\Sigma-\Sigma_\delta}j_z(s)\left(\frac{e^{ikR}}{R}\right)d\Sigma \tag{S28}$$

where $R=\sqrt{4R_{CN}^2\sin^2\left(\frac{\phi}{2}\right)+(s-z')^2}$.

Thus,

$$\frac{\partial \nu(z')}{\partial z'} = \lim_{\delta\to 0}\int_{\Sigma-\Sigma_\delta} j_z(s)\frac{\partial}{\partial z'}\left(\frac{e^{ikR}}{R}\right)d\Sigma =$$

$$-\lim_{\delta\to 0}\int_{\Sigma-\Sigma_\delta} j_z(s)\frac{\partial}{\partial s}\left(\frac{e^{ikR}}{R}\right)d\Sigma =$$

$$\lim_{\delta\to 0}\left[\int_{\Sigma-\Sigma_\delta}\frac{\partial j_z(s)}{\partial s}\left(\frac{e^{ikR}}{R}\right)d\Sigma - \int_{\Sigma-\Sigma_\delta}\frac{\partial}{\partial s}\left(j_z(s)\frac{e^{ikR}}{R}\right)d\Sigma\right] = \tag{S29}$$

$$\lim_{\delta\to 0}\left[\int_{\Sigma-\Sigma_\delta}\frac{\partial j_z(s)}{\partial s}\left(\frac{e^{ikR}}{R}\right)d\Sigma - \int_C j_z(s)\frac{e^{ikR}}{R}dC\right]$$

where the integral over C_δ in the last term vanishes. Performing the second derivative gives,

$$\frac{\partial^2 \nu(z')}{\partial z'^2} = \lim_{\delta\to 0}\left[\int_{\Sigma-\Sigma_\delta}\frac{\partial\left(j_z(s)-j_z(z')\right)}{\partial z'}\frac{\partial}{\partial z'}\left(\frac{e^{ikR}}{R}\right)d\Sigma - \int_C j_z(s)\frac{\partial}{\partial z'}\left(\frac{e^{ikR}}{R}\right)dC\right] =$$

$$\lim_{\delta\to 0}\left[\int_{\Sigma-\Sigma_\delta}\frac{\partial\left(j_z(s)-j_z(z')\right)}{\partial z'}\frac{\partial^2}{\partial z'^2}\left(\frac{e^{ikR}}{R}\right)d\Sigma + \int_C\left(j_z(s)-j_z(z')\right)\frac{\partial}{\partial z'}\left(\frac{e^{ikR}}{R}\right)dC\right] \tag{S30}$$

Finally, by using

$$\lim_{\delta\to 0}\int_{\Sigma-\Sigma_\delta}\left(j_z(s)-j_z(z')\right)\frac{\partial^2}{\partial z'^2}\left(\frac{e^{ikR}}{R}\right)d\Sigma = \tag{S31}$$

$$= V.P.\int_{-L}^{L}\frac{\partial^2}{\partial s^2}\left(\int_0^{2\pi}\frac{e^{ikR}}{R}d\phi\right)\left(j_z(s)-j_z(z')\right)dz'$$

and

$$\int_C\left(j_z(s)-j_z(z')\right)\frac{\partial}{\partial z'}\left(\frac{e^{ikR}}{R}\right)dC = \tag{S32}$$

$$= j_z(z')\left(\left.\int_0^{2\pi}\frac{e^{ikR}}{R}d\phi\right|_{z'=-L} - \left.\int_0^{2\pi}\frac{e^{ikR}}{R}d\phi\right|_{z'=L}\right)$$

we obtain

$$\frac{\partial^2 \nu(z')}{\partial z'^2} = V.P.\int_{-L}^{L}\frac{\partial^2 G(z'-s)}{\partial s^2}\left(j_z(s)-j_z(z')\right)ds +$$

$$j_z(z')\frac{\partial}{\partial z'}\left(G(z'-L)-G(z'+L)\right) \tag{S33}$$

Multiplying (S33) by $g(z,z')$ and integrating over the CNT surface, one gets

$$\int_{-L}^{L} \frac{\partial^2 \nu(z')}{\partial z'^2} g(z,z') dz' = V.P.\int_{-L}^{L} j_z(s) \int_{-L}^{L} \frac{\partial^2 G(z'-s)}{\partial s^2} g(z,z') dz' ds -$$
$$-V.P.\int_{-L}^{L} j_z(z') \int_{-L}^{L} \frac{\partial^2 G(z'-s)}{\partial s^2} g(z,z') ds dz' + \int_{-L}^{L} j_z(z') \frac{\partial}{\partial z'} \big(G(z'-L) - G(z'+L)\big) dz' \tag{S34}$$

Note that in the second term on the right hand side of (S34), we have exchanged $\partial^2/\partial s^2 \to \partial^2/\partial z'^2$, $s \to z', z' \to s$, and $z' \to s$ in the third term. As a result, we can express the kernel in a convenient form as

$$K(z,s) = \left(\frac{\partial^2}{\partial s^2} + k^2\right) \int_{-L}^{L} g(z,z') G(s-z') dz' = F_1(z,s) - F_2(z,s) + F_3(z,s) \tag{S35}$$

$$F_1(z,s) = k^2 \text{V.P.} \int_{-L}^{L} g(z,z') G(s-z') dz' \tag{S36}$$

$$F_2(z,s) = g(z,s) \frac{\partial}{\partial s} \big(G(s-L) - G(s+L)\big) \tag{S37}$$

$$F_3(z,s) = \text{V.P.} \int_{-L}^{L} \big(g(z,z') - g(z,s)\big) \frac{\partial^2 G(s-z')}{\partial s^2} dz' \tag{S38}$$

where the symbol V.P. denotes the Cauchy principal value of the integral. This is one of the kernel representations used in our numerical simulations

Representation based on the addition theorem for Hankel functions

Expanding the Green function of the Helmholtz equation by cylindrical modes, yields

$$\frac{e^{ik\sqrt{r^2+R_{CN}^2-2rR_{CN}\sin(\phi)+(s-z)^2}}}{\sqrt{r^2+R_{CN}^2-2rR_{CN}\sin(\phi)+(s-z)^2}} = \frac{i}{2} \int_{-\infty}^{\infty} e^{ih(z-s)} H_0^{(1)}\left(\sqrt{k^2-h^2}\,\bar{r}\right) dh \tag{S39}$$

where $\bar{r} = \sqrt{r^2 + R_{CN}^2 - 2rR_{CN}\sin(\phi)}$. The addition theorem for Hankel functions gives

$$H_0^{(1)}(\kappa \bar{r}) = \sum_n H_{-n}^{(1)}(\kappa r) J_n(\kappa R_{CN}) e^{in\phi} \tag{S40}$$

where $r > R_{CN}$. Substituting (S39) and (S40) in Equation (12), integrating over the angle ϕ ,keeping only one term with $n = 0$, gives

$$G(z-s) = G(R_{CN}, z-s) = i\pi R_{CN} \int_{-\infty}^{\infty} e^{ih(z-s)} H_0^{(1)}(\kappa R_{CN}) J_0(\kappa R_{CN}) dh \tag{S41}$$

where $\kappa = \sqrt{k^2 - h^2}$. Restricting the sign of $\text{Im}\left(\sqrt{k^2-h^2}\right)$ is imposed to assure the proper decay of the Hankel function as $|h| \to \infty$. As a next step, we substitute (S40) in Eqs. (S36) - (S38), which leads to

$$K(z,s) = i\pi R_{CN}\left(\frac{\partial^2}{\partial s^2}+k^2\right)\int_{-\infty}^{\infty} e^{ihs}H_0^{(1)}(\kappa R_{CN})J_0(\kappa R_{CN})\left(\int_{-L}^{L} g(z,z')e^{-ihz'}dz'\right)dh \tag{S42}$$

where $g(z,z')$ is the Green function given in (S21). An integration over z' readily yields

$$\int_{-L}^{L} g(z,z')e^{-ihz'}dz' = -\frac{F(h,z)}{(\tilde{\alpha}^2-h^2)\sin(2\tilde{\alpha}L)} \tag{S43}$$

where $F(h,z) = e^{-ihz}\sin(2\tilde{\alpha}L)+e^{ihL}\sin X - e^{-ihL}\sin Y$, and $X=\tilde{\alpha}(z-L), Y=\tilde{\alpha}(z+L)$. The right hand side of (S42) is nonsingular at $h=\pm\tilde{\alpha}$, since $F(\pm\tilde{\alpha},z)=0$. Note that this function also satisfies the following symmetry relation $F(h,z)=F(-h,-z)$, which implies that $K(z,s)=K(-z,-s)$ .Finally, by changing the order of integration over h in (S42), and substituting (S43) in (S42), we obtain

$$K(z,s) = -\frac{i\pi R_{CN}}{\sin(2\tilde{\alpha}L)}\int_{-\infty}^{\infty} e^{ihs}\left(k^2-h^2\right)\frac{H_0^{(1)}(\kappa R_{CN})J_0(\kappa R_{CN})}{\kappa^2-k^2}F(z,h)dh \tag{S44}$$

This is an another form of the kernel, which is equivalent to the form of equation (S34) that leads to the same numerical results.

Comparison of the two kernel representations

To assess the numerical implementation of the two alternative representations of the kernel, we independently solved the integral equation using the singular-integral representation of Equation (S34) and the representation based on the addition theorem for Hankel functions, Equation (S43). The resulting real and imaginary parts of the axial surface-current density ($j_z(z)$) are compared in Figure 1S. Both formulations predict the same qualitative spatial dependence of the induced current. In particular, the current is nearly uniform over most of the CNT length and exhibits pronounced variations in the vicinity of the two terminations. The two solutions also preserve the expected symmetry with respect to the CNT center. Nevertheless, a systematic quantitative difference between the two numerical evaluations is observed. The difference is approximately uniform over the interior of the CNT, whereas larger local deviations occur close to the ends, where the current varies rapidly. Since Equations (S34) and (S43) are derived as alternative representations of the same kernel, the observed discrepancy should be attributed to their numerical evaluation rather than to a difference in the underlying physical model. In particular, the singular-integral formulation requires the numerical treatment of Cauchy principal-value integrals, whereas the addition-theorem representation involves integration over the spectral variable (h). Consequently, the two formulations have different numerical convergence properties and may require different discretization and truncation parameters. The inserts show the detailed current distribution in the end vicinities. One can see physically correct physical behavior of the current at the ends for both types of kernel presentation.

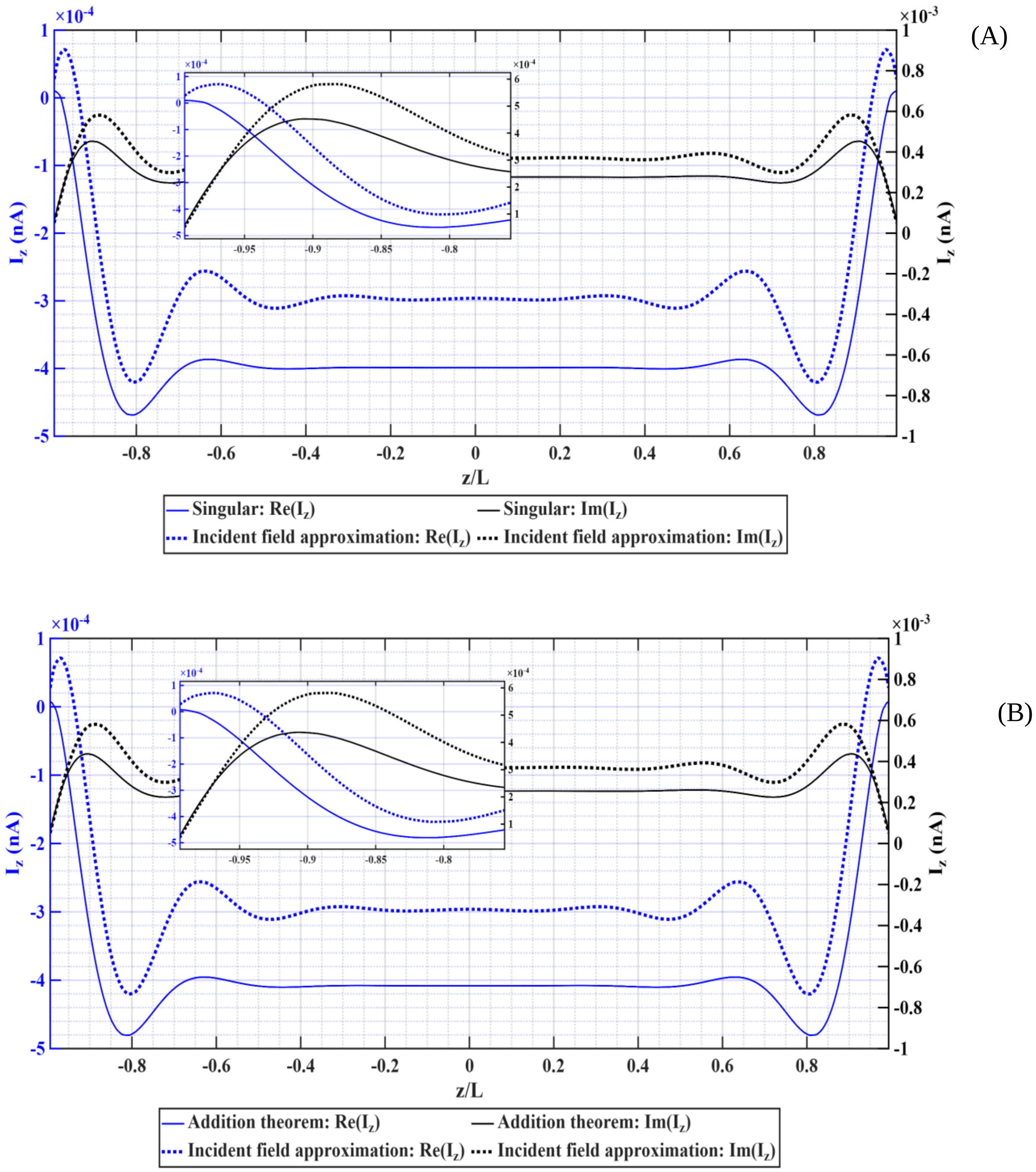

Figure S1. Comparison between the two equivalent kernel representations. Real (A) and imaginary (B) parts of the induced axial surface current $j_z(z)$ as functions of the normalized axial coordinate (*z/L*), calculated using the kernel representation based on the addition theorem for Hankel functions (blue solid line) and the singular-integral representation (red dashed line). The CNT extends over *(-L, L)*, such that (*L*) denotes its half-length. Both formulations reproduce the same characteristic spatial structure of the induced current, including an approximately uniform response over the central portion of the CNT and pronounced variations near the two terminations. The calculations are performed for a zigzag (12,0) CNT with radius $R_{CN} = 0.47\text{nm}$, using an electrochemical potential $\mu = 0.4$ eV at T=300K, incident electric-field of amplitude $E_z^{inc} = 1.0\,\text{V/m}$ and frequency 200THz impinging the tube with an incidence angle $\theta_0 = 90^{\circ}$. The remaining electromagnetic parameters are identical for the two calculations, so that the differences shown arise solely from the numerical implementation of the two mathematically equivalent kernel representations.

References

[S1]. Katsnelson, M.I. (2020). *The Physics of Graphene*, Second Edition, Cambridge University Press.

[S2]. Berghaus, T., Miloh, T., Gottlieb, O., and Slepyan, G.Y. (2025). Electrodynamics of carbon nanotubes with non-local surface conductivity. *Appl. Sci.* **15**, 11398. doi.org/10.3390/app152111398.

[S3]. Falkovsky, L.A., and Pershoguba, S.S. (2007). Optical far-infrared properties of a graphene monolayer and multilayer. *Phys. Rev. B* **76**, 153410. doi.org/10.1103/PhysRevB.76.153410.

[S4]. Slepyan, G.Y., Maksimenko, S.A., Lakhtakia, A., Yevtushenko, O., and Gusakov, A.V. (1999). Electrodynamics of carbon nanotubes: Dynamic conductivity, impedance boundary conditions, and surface wave propagation. *Phys. Rev. B* **60**, 17136. doi.org/10.1103/PhysRevB.60.17136.

[S5]. Forati, E., Mueller, A.D., Gandomkar Yarandi, P., and Hanson, G.W. (2011). A new formulation of Pocklington's equation for thin wires using the exact kernel. *IEEE Trans. Antennas Propag.* **59**, 4355–4360. doi.org/10.1109/TAP.2011.2164211.
